# A systematic review of neurological symptoms and complications of COVID-19

**DOI:** 10.1007/s00415-020-10067-3

**Published:** 2020-07-20

**Authors:** Xiangliang Chen, Sarah Laurent, Oezguer A. Onur, Nina N. Kleineberg, Gereon R. Fink, Finja Schweitzer, Clemens Warnke

**Affiliations:** 1 Department of Neurology, Nanjing First Hospital, Nanjing Medical University, Nanjing, China; 2 Department of Neurology, Faculty of Medicine and University Hospital Cologne, University of Cologne, Kerpener Street 62, 50937 Cologne, Germany; 3 Cognitive Neuroscience, Institute of Neuroscience and Medicine (INM-3), Research Centre Jülich, Leo-Brandt-Strasse, Jülich, 52425 Germany

**Keywords:** SARS-CoV-2, COVID-19, Neuro-COVID, Nervous system

## Abstract

**Objective:**

To study the frequency of neurological symptoms and complications in COVID-19 patients in a systematic review of the literature.

**Methods:**

Relevant studies were identified through electronic explorations of PubMed, medRxiv, and bioRxiv. Besides, three Chinese databases were searched. A snowballing method searching the bibliographies of the retrieved references was applied to identify potentially relevant articles. Articles published within 1 year prior to April 20th, 2020, were screened with no language restriction imposed. Databases were searched for terms related to SARS-CoV-2/COVID-19 and neurological manifestations, using a pre-established protocol registered on the International Prospective Register of Systematic Reviews database (ID: CRD42020187994).

**Results:**

A total of 2441 articles were screened for relevant content, of which 92 full-text publications were included in the analyses of neurological manifestations of COVID-19. Headache, dizziness, taste and smell dysfunctions, and impaired consciousness were the most frequently described neurological symptoms, the latter more often among patients with a severe or critical disease course. To date, only smaller cohort studies or single cases have reported cerebrovascular events, seizures, meningoencephalitis, and immune-mediated neurological diseases, not suitable for quantitative analysis.

**Conclusion:**

The most frequent neurological symptoms reported in association with COVID-19 are non-specific for the infection with SARS-CoV-2. Although SARS-CoV-2 may have the potential to gain direct access to the nervous system, so far, SARS-CoV-2 was detected in the cerebrospinal fluid in two cases only. Standardized international registries are needed to clarify the clinical relevance of the neuropathogenicity of SARS-CoV-2 and to elucidate a possible impact of SARS-CoV-2 infection on common neurological disease, such as Alzheimer’s, Parkinson’s disease or multiple sclerosis.

**Electronic supplementary material:**

The online version of this article (10.1007/s00415-020-10067-3) contains supplementary material, which is available to authorized users.

## Introduction

The rapidly evolving coronavirus disease 2019 (COVID-19) pandemic is caused by the severe acute respiratory syndrome coronavirus 2 (SARS-CoV-2) [1]. In COVID-19 patients, neurological manifestations such as impaired consciousness, stroke, and seizure have been reported, with a higher incidence in those with a more severe course of COVID-19 [2]. However, these manifestations not necessarily require direct infection of the peripheral (PNS) or the central nervous system (CNS), but could also occur secondary to a severe systemic reaction in response to a viral infection outside the nervous system. In the past months, reports of meningitis, encephalitis, myelitis, or peripheral nerve affection in the context of COVID-19 were published, suggesting that SARS-CoV-2 can directly infect structures of the nervous system. However, a systematic review that differentiates between neurological symptoms occurring during systemic viral infections in general from SARS-CoV-2-specific neurological complications has not been published.

**Fig. 1:**
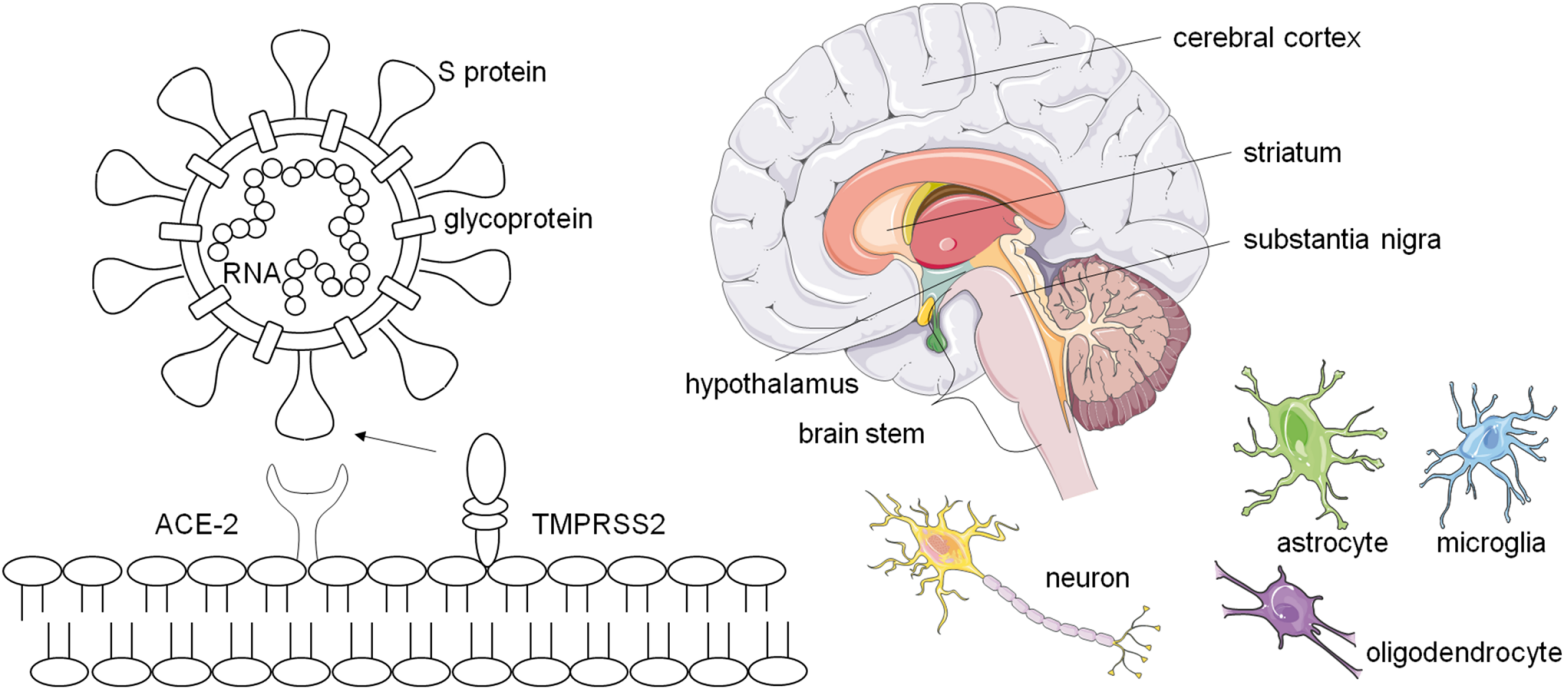
Neurotropism of SARS-CoV-2. SARS-CoV-2 spike (S) proteins bind angiotensin-converting enzyme 2 (ACE-2) receptor of target cell. Cleavage of the S protein by type II transmembrane serine protease (TMPRSS2), facilitates viral entry. ACE-2 mRNA expression and double-positive ACE-2 + TMPRSS2 + cells have been identified, amongst others, on neurons and glial cells, in the cerebral cortex, striatum, hypothalamus, substantia nigra and brain stem, making the CNS potential direct targets of SARS-CoV-2 infection ( Adapted from Servier Medical Art, https://smart.servier.com)

**Fig. 2:**
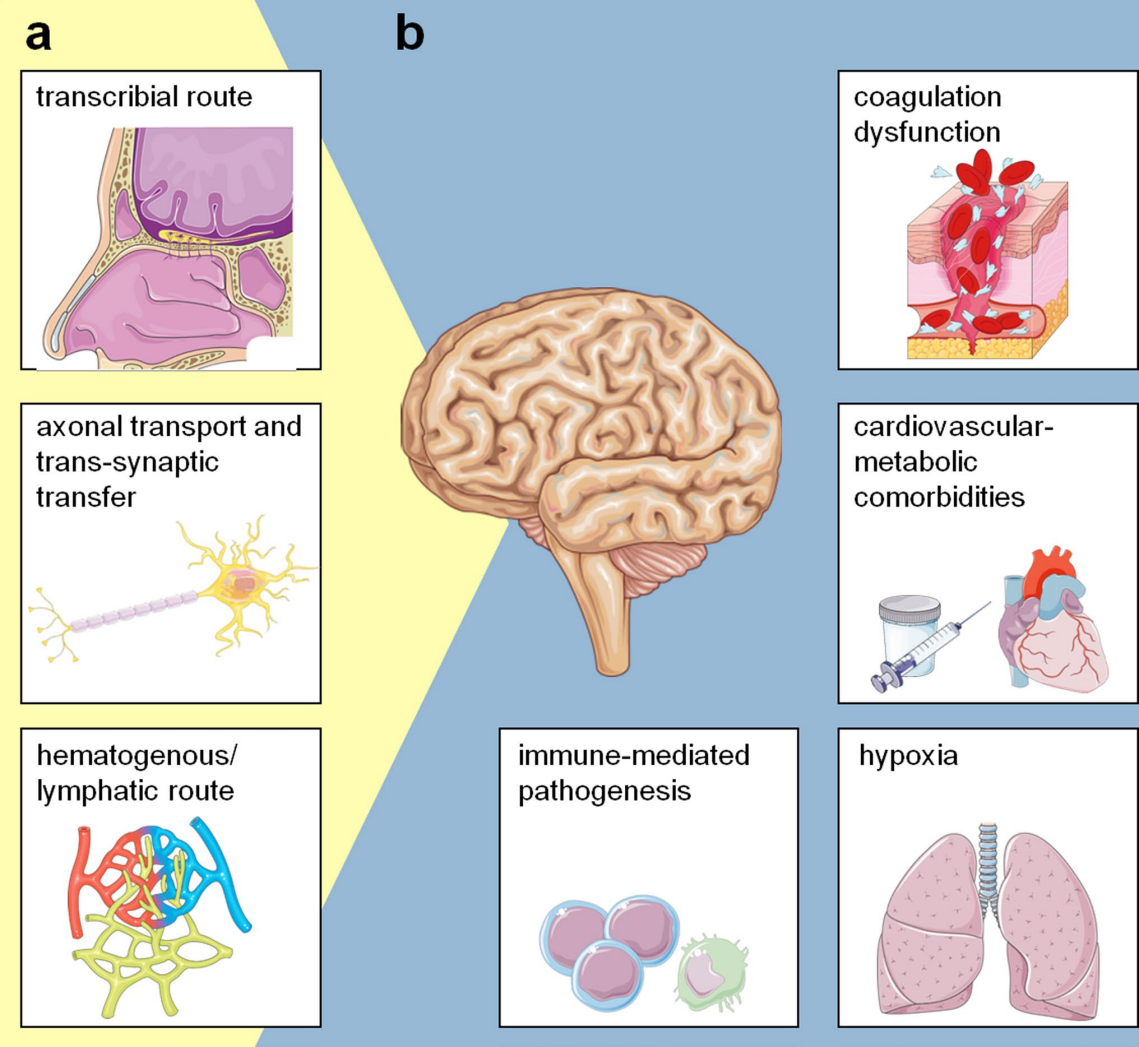
Neuropathogenesis of SARS-CoV-2. **a** Three potential mechanisms of SARS-CoV-2 invasion into the CNS. (1) CNS entry through the transcribial route, involving infection of the olfactory epithelium [13], (2) axonal transport and trans-synaptic transfer, including infection of various peripheral nerve terminals [17] and the spread along nerves [15] and (3) viral spread through the bloodstream or lymphatic system [18]. **b** Factors indirectly influencing neurotoxicity. Immune-mediated pathogenesis, associated with, amongst others, lymphocytopenia [2] and T-helper 1 cell-mediated neuroinflammation [20, 21] coagulation dysfunction including higher D-dimer levels, prolonged prothrombin time, and decreased platelet counts [22], as well as hypoxia [26], disturbances of the gut microbiome during gastrointestinal SARS-CoV-2 infection [27] and cardiovascular-metabolic comorbidities like hypertension, diabetes [23] and altered glucose and lipid metabolism [24, 25] might all influence SARS-CoV-2 neuropathogenicity ( Adapted from Servier Medical Art, https://smart.servier.com)

The SARS-CoV-2 spike (S) protein can bind to the host cellular angiotensin-converting enzyme 2 (ACE-2) receptor, which is of relevance to cell tropism [3, 4]. Processing and priming of the S protein by the transmembrane protease serine 2 (TMPRSS2) have been shown to be essential for the fusion of viral and host cellular membranes and entry of SARS-CoV-2 [5]. The ACE-2 receptor expression has recently been found on neurons and glial cells of several brain structures [6, 7], including the cerebral cortex, the striatum, the posterior hypothalamic area, the substantia nigra, and brain stem [8–11] (Fig. 1). While systematic and experimental studies regarding the neurotropism of SARS-CoV-2 are lacking [12], several mechanisms such as the transcribial route [13, 14], the axonal transport and trans-synaptic transfer [15–17], and the hematogenous and/or lymphatic route [18] are currently discussed as possible viral access routes to the brain. The invasion of the CNS via the transcribial route describes an infection of the olfactory epithelium and successional transmission through the cribriform plate to the subarachnoid space. In contrast, the axonal transport and trans-synaptic transfer would include the infection of various (peripheral) nerve terminals and a spreading along neurons, such as the olfactory bulb, the trigeminal nerve, or the vagus nerve in the respiratory or gastrointestinal tract, respectively [16, 17, 19]. A third route postulates a CNS invasion by SARS-CoV-2 through the bloodstream or the lymphatic system [18]. Migration across the brain endothelium could be achieved by direct infection of brain microvascular endothelial cells (BMEC) and abluminal virus release into the CNS parenchyma [17], or by endocytosis, via virally infected leukocytes or disrupted tight junctions on BMECs. All these pathways may lead to neurological affection by direct infection of the PNS or CNS (Fig. 2a).

Notably, not all neurological symptoms or complications require direct infection of cells or structures of the nervous system. Indirect neurotoxicity may result secondary to immune-mediated pathogenesis [2, 20, 21], coagulation dysfunction [22], cardiovascular comorbidities like hypertension or diabetes [23], altered glucose and lipid metabolism [24, 25], disturbances in the lung-brain cross talk such as hypoxic encephalopathy [26], or as a consequence of an imbalanced gut-brain axis through disturbances of the gut microbiome during gastrointestinal SARS-CoV-2 infection [27] (Fig. 2b).

This systematic review summarizes the available data on neurological symptoms and complications in patients with COVID-19, categorizing the findings into frequently occurring symptoms or rare neurological complications.

## Methods

### Protocol and registration

This systematic review was conducted following a pre-established protocol registered on the International Prospective Register of Systematic Reviews (PROSPERO) database (ID: CRD42020187994). It is reported according to the PRISMA (Preferred Reporting Items for Systematic Reviews and Meta-Analyses) guidelines.

### Eligibility criteria

Articles that reported neurological manifestations of COVID-19, including epidemiological, clinical, laboratory, or radiological features, as well as neurotropism and neuropathogenesis, were considered for inclusion.

Eligible study designs were observational studies that involved case reports, case series, case–control studies, cohort studies, and letters. Literature reviews with the respective reference lists were screened. Editorials were excluded. Studies with less than 30 subjects were excluded if they only reported non-specific neurological symptoms. For observations of rare but severe neurological manifestations, no study size restrictions were applied. Reviews, viewpoints or animal and in vitro studies were only included to explain putative neurotropic mechanisms described in the introduction.

### Search strategy

Relevant studies were identified through electronic searches of PubMed, medRxiv, and bioRxiv, as well as three Chinese databases: China National Knowledge Infrastructure, WanFang, and the Chinese Medical Journal Network. Besides, a snowballing method searching the bibliographies of retrieved references was applied to identify potentially relevant articles. Articles were screened within 1 year prior to April 20th, 2020. No language restrictions were imposed. Databases were searched for terms related to (1) SARS-CoV-2 or (2) COVID-19 AND (3) neurological aspects. The following search terms were used in PubMed: (“Wuhan coronavirus” or “Wuhan virus” or “novel coronavirus” or “nCoV” or “SARS-CoV-2” or “SARS 2” or “severe acute respiratory syndrome coronavirus 2” or “COVID-19” or “coronavirus disease 2019 virus” or “2019-nCoV” or “2019 novel coronavirus” or “severe acute respiratory syndrome coronavirus 2” or “coronavirus” or “coronaviruses”) AND (“nervous system” or “CNS” or “brain” or “neural” or “neuro*” or “encephalitis” or “myelitis” or “headache” or “dizziness” or “anosmia” or “ageusia” or “consciousness” or “cerebral” or “cerebrovascular” or “seizure” or “epilepsy” or “Guillain-Barré syndrome” or “Miller Fisher Syndrome”).

### Study selection

Two reviewers first screened titles and abstracts of all retrieved records for duplication (X.C. and S.L.). Full texts of all potentially relevant studies were then independently studied to determine the final study selection. Discrepancies were resolved by consensus. Duplicate information on the same patients was combined to obtain complete data.

### Data extraction

The following data were extracted from eligible articles: study characteristics (study title, authors, date of publication, publication type, study site, number of subjects), population characteristics, and the association with neurological disease. Data were extracted independently by three authors (S.L., F.S., and X.C.). A fourth author (C.W.) resolved discrepancies in data extraction and checked the retrieved information to rule out duplication. For quality assessment, two authors (X.C. and F.S.) independently assessed (1) the criteria for the diagnosis of COVID-19, (2) the laboratory confirmation method of SARS-CoV-2, and (3) the respiratory specimens used for testing.

### Data synthesis

Descriptive analyses were applied as most of the featured studies were case reports. Percentages and 95% confidence intervals (generated using a web CI calculator for single incidence rate [28]) were calculated. Frequent neurological manifestations were defined to have a frequency of at least 4% and reported in at least five different studies. The term “rare neurological manifestations” was applied when only a few cases of relevant findings were reported, e.g., in case reports or smaller cohort studies.

## Results

### Identification of relevant study data

**Fig. 3:**
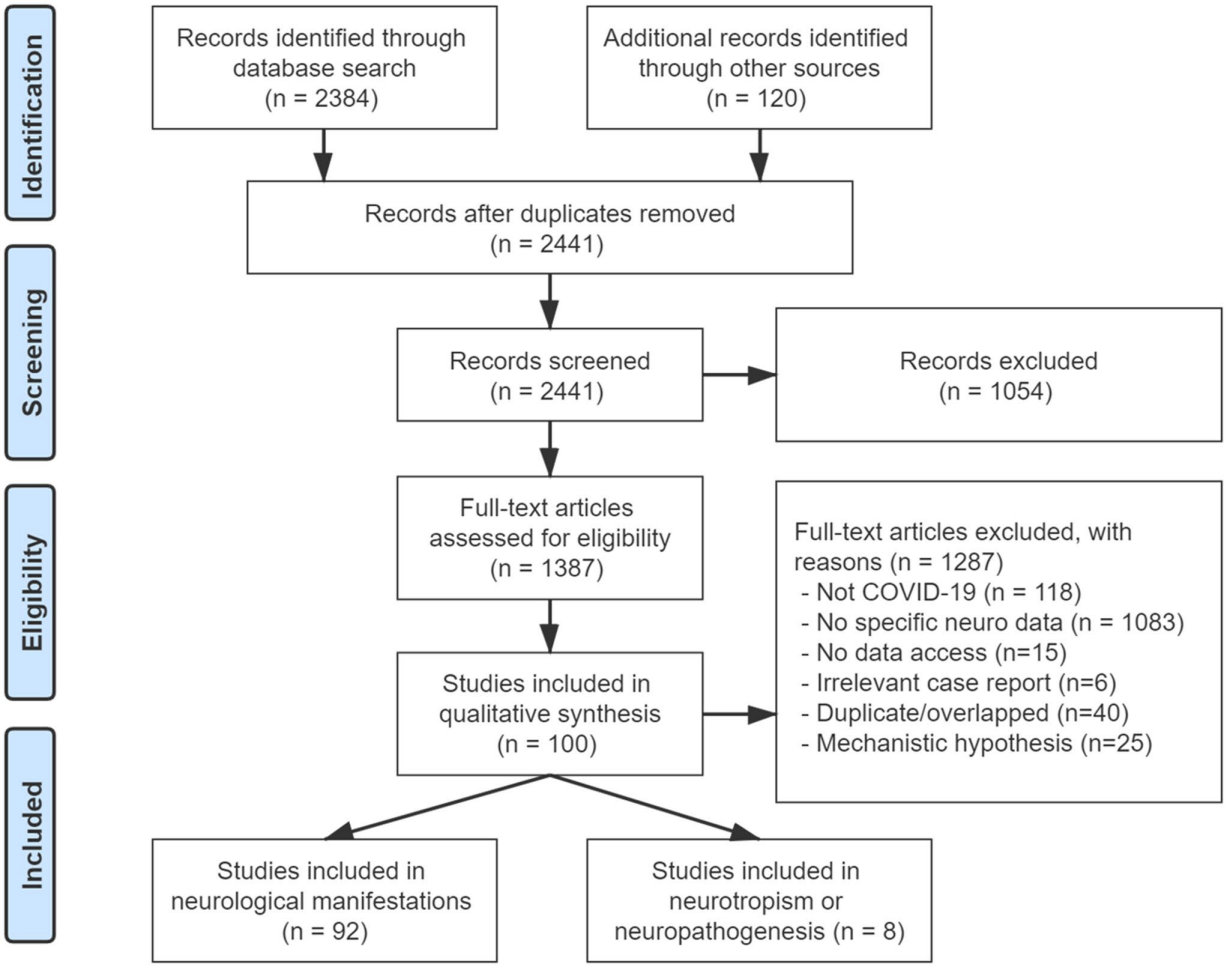
Study identification PRISMA flow diagram

A total of 2441 articles were screened for eligible content, of which 1387 full-text publications were assessed. The majority was excluded (*n* = 1287), mainly due to non-relevance for the investigated topic. A total of eight studies on mechanisms of neurotropism and neuropathogenesis were included to generate Figs. 1 and 2 of “Introduction”. Ninety-two studies were included for analyzing the frequency of neurological manifestations of COVID-19 (Fig. 3).

### Frequent neurological manifestations of COVID-19

Headache, dizziness, taste and smell dysfunctions, or impaired consciousness were the most frequently reported neurological symptoms in COVID-19 patients, each observed in more than five of the analyzed studies, and with an overall frequency of greater than 4% of the populations studied (Table 1).

**Table 1:** Summary of frequent neurological symptoms reported in COVID-19 patients

	Total			Mild or moderate^a^			Severe or critical^b^		
	*n* studies	*n*/*N* (%)^c^	95% CI	*n* studies	*n*/*N* (%)^c^	95% CI	*n* studies	*n*/*N* (%)^c^	95% CI
Headache	51	3308/16,446 (20.1)	19.5—20.7	25	432/4007 (10.8)	9.9—11.8	24	161/1937 (8.3)	7.2—9.6
Dizziness	13	151/2236 (6.8)	5.8—7.9	6	54/839 (6.4)	5.0—8.3	5	45/590 (7.6)	5.8—10.1
Headache or dizziness	8	79/654 (12.1)	9.8—14.9	5	45/403 (11.2)	8.5—14.7	5	8/97 (8.2)	4.2—16.0
Smell dysfunction^d^	6	536/906 (59.2)	56.0—62.4	3	380/585 (65.0)	61.2—68.9	1	3/88 (3.4)	1.1—10.4
Taste dysfunction^d^	6	430/846 (50.8)	47.6—54.3	3	365/553 (66.0)	62.2—70.1	1	3/88 (3.4)	1.1—10.4
Impaired consciousness	9	146/2890 (5.1)	4.3—5.9	3	19/597 (3.2)	2.1—5.0	4	72/605 (11.9)	9.6—14.8

Data are represented as a total of all studies reporting symptoms and classified according to disease severity when reported

Severity was defined with ^a^mild or moderate meaning clinical symptoms with or without imaging findings of pneumonia, and ^b^severe or critical if they have the following: respiratory rate > 30 breaths/min, severe respiratory distress, or SpO_2_ ≤ 93% on room air, PaO_2_/FiO_2_ ≤ 300 mmHg, or have one of the following: respiratory failure requiring mechanical ventilation, shock, and other organ failure needing ICU treatment

^c^Data reported as *n*/*N* (%), where *N* is the total number of patients studied and *n* the number of patients showing symptoms

^d^One study was excluded from this table, in which smell and taste impairments were not reported separately

### Headache and dizziness

Headache was assessed in 51 studies, involving 16,446 COVID-19 patients. Of these, headache was reported in 20.1% of the population studied, ranging from 2.0 [29] to 66.1% [30] (Online Resource Table III). In patients with COVID-19 and available data on the severity of disease course, headache was reported more frequently in mild or moderate compared to severe or critical disease (10.8% vs. 8.3%, 95% CI not overlapping).

Dizziness was investigated in 13 studies, including 2236 COVID-19 patients. Approximately 7.0% (ranging from 2.5 [31] to 21.4% [32]) of the COVID-19 patients were reported to have suffered from dizziness, equally reported among mild or moderate compared with severe or critical cases. Further clinical distinction between dizziness and vertigo as well as data regarding the etiology of these symptoms were not reported in the included studies. Therefore, the underlying cause of dizziness (i.e. general weakness, neuropathy, involvement of the eighth cranial nerve, stroke) remain unclear.

Eight studies, involving 654 COVID-19 patients, reported headache or dizziness as a combined manifestation, occurring in 12.1%, with no difference for mild or moderate vs. severe or critical disease courses.

### Smell and taste dysfunction

Various reports concerning smell (*n* = 6, including 906 patients) and taste dysfunctions (*n* = 6, including 846 patients) have been published, with high variation in the reported frequency (Table 1). While one study noted impaired smell and taste in 5.1% and 5.6% of the patients, respectively [2], a larger study in 417 patients with mild to moderate SARS-CoV-2 infection noted smell dysfunction in 85.6% and taste dysfunction in 88.8% of patients [33]. In most of the cases, smell dysfunction appeared after (65.4%) or simultaneously (22.8%) with general or ear, nose, throat symptoms [33]. Across the studies, smell dysfunction was reported in 59.2% and taste dysfunction in 50.8% of patients. Both were more frequently reported in COVID-19 patients with mild or moderate (65.0% and 66.0%, respectively) as compared to severe or critical disease courses (3.4%, 95% CI not overlapping).

### Impaired consciousness

Overall, nine studies, including 2890 patients, reported impairment of consciousness (also termed “confusion” or “agitation”) in 5.1% of COVID-19 patients (Table 1), ranging from 1.4 [34] to 69.0% [35] of the patients. As expected, impaired consciousness was noted more frequently in severe or critical compared with mild or moderate COVID-19 patients (11.9% vs. 3.2%, 95% CI not overlapping) and in non-survivors compared with survivors (21.2%, 95% CI 15.0–30.0 vs. 1.1%, 95% CI 0.5–2.3).

### Rare neurological manifestations of COVID-19

Few reports of severe neurological manifestations have been published. These smaller cohort studies or case reports only allowed a descriptive summary, given in Online Resource Table II and the following section of this review.

### Acute cerebrovascular complications

Acute cerebrovascular events in COVID-19 patients were reported in two cohort studies. Mao et al. reported that 2.8% (6 out of 214 hospitalized patients) developed acute cerebrovascular events, of which the vast majority (5 of the 6 cases) had a severe or critical disease course [2]. In a retrospective observational analysis including 221 COVID-19 patients, Li et al. detected 11 patients with acute ischemic stroke, one with cerebral venous sinus thrombosis, and one with cerebral hemorrhage among COVID-19 patients. Patients, who developed acute cerebrovascular events were significantly older (71.6 ± 15.7 years vs. 52.1 ± 15.3 years), more likely to present with severe COVID-19 (84.6% vs. 39.9%), and also more likely to present with cardiovascular risk factors, including hypertension (69.2% vs. 22.1%), diabetes (46.2% vs. 12.0%), and previous medical history of cardio-cerebrovascular diseases (23.1% vs. 6.7%) [36]. Two further case reports described COVID-19 patients suffering from cerebral infarctions [37, 38].

### Seizures

Generalized seizures were reported in two case reports of COVID-19 patients [39, 40]. However, CSF and cerebral MRI analyses were not performed in one of these patients, leaving insecurity about diagnostic accuracy [40]. Neither acute symptomatic seizures, nor status epilepticus were observed in a more extensive retrospective study involving 304 COVID-19 patients [41].

### Meningitis/encephalitis

Seven single-case reports on meningitis/encephalitis in association with COVID-19 have been published. In two of these patients, CSF was positive for SARS-CoV-2: One case of viral encephalitis was reported from China with only minimal clinical details provided [42]. Another case of encephalitis was reported in a patient from Japan [39], presenting with altered consciousness, generalized seizure, and positive SARS-CoV-2 PCR in CSF, as well as a pathological cerebral MRI (right lateral ventriculitis and encephalitis mainly on the right mesial temporal lobe and hippocampus). In three further cases, SARS-CoV-2 RNA was not detected in CSF: in one case from China, a cerebral CT was normal, but no MRI was performed, thus again, leaving uncertainty in the diagnosis of encephalitis [43]. A case of COVID-19 with tuberculous meningitis was also reported from China, with CSF positive for mycobacterium tuberculosis and negative for SARS-CoV-2, and a pathological cerebral CT [44]. The third case, from Italy, showed an unremarkable cerebral CT and MRI (including with gadolinium); the EEG showed generalized slowing [45]. For the remaining two cases, no SARS-CoV-2 CSF testing was performed. One case of acute hemorrhagic necrotizing encephalopathy was reported from the US. MRI showed hemorrhagic rim enhancing lesions within the thalamus bilaterally, the medial temporal lobes, and subinsular regions [46]. Another case from the US showed a normal cerebral CT and a generalized slowing in the EEG [40], but no epileptic discharges.

Finally, a case of flaccid myelitis, in which neither CSF nor MRI analyses were performed, was reported [47] (Online Resource Table II).

### Guillain–Barré syndrome (GBS), Miller Fisher syndrome, and polyneuritis cranialis

Zhao et al. [48] reported a patient with GBS, who developed symptoms of COVID-19 during an in-hospital stay, suggesting a co-incidental association. Gutiérrez-Ortiz [49] described one case of Miller Fisher syndrome (with positive antibodies against GD1b-IgG) and a case of polyneuritis cranialis. In both patients, SARS-CoV-2 PCR in CSF was negative.

### Oculomotor nerve palsy

One case of oculomotor nerve palsy was reported in a COVID-19 patient. The cerebral MRI was not conclusive, and SARS-CoV-2 was not detected in CSF [50].

## Discussion

The COVID-19 pandemic is one of the biggest medical challenges of this century. The wealth of medical data generated is contrasted by the dearth of data on the frequency of neurological symptoms and occurrence of (rare) neurological complications.

As summarized above, headache, dizziness, and impaired consciousness are neurological symptoms frequently observed in patients with COVID-19. Such symptoms are not specific for infection with SARS-CoV-2 and may also be found in other viral infections. These symptoms do not necessarily postulate an infection of underlying neurological structures, but could also occur via indirect mechanisms of neuropathogenicity, e.g., as a consequence of respiratory distress, hypoxia, or due to hypotonia, dehydration, and fever during sepsis. Indirect mechanisms of neuropathogenicity may be sufficient to explain headache and dizziness as frequent non-specific symptoms in mild or moderate, as well as impaired consciousness in severe or critical COVID-19 patients. The latter might be confounded by the fact that impaired consciousness is frequently noted in hospitalized elderly patients.

Interestingly, a high frequency of olfactory and gustatory dysfunction in COVID-19 patients has been noted. A loss of olfactory function in viral infections is well known in otolaryngology. Viruses such as rhinovirus, parainfluenza, Epstein–Barr virus, and some other CoVs may cause olfactory dysfunction through an inflammatory reaction within the nasal mucosa and the occurrence of rhinorrhea [51, 52]. Data published by Lechien and colleagues suggest, however, that olfactory dysfunction associated with COVID-19 infection may appear in the absence of rhinorrhea [33]. Therefore, nasal inflammation and related obstruction may not be the only etiological factors underlying the frequent observation of smell and taste dysfunctions in patients with COVID-19. Indeed, the transcribial route has been suggested as one possible route of SARS-CoV-2 to the brain and its direct infection.

Yet, data on direct brain infection by SARS-CoV-2 are very limited. Moriguchi et al. and Poyiadji et al. described the most compelling cases of encephalitis with the detection of SARS-CoV-2 RNA in CSF, constituting strong evidence for neurotropism [39]. Notwithstanding, in most reports on encephalitis and related disorders, SARS-CoV-2 RNA was neither detected in CSF, nor relevant further examinations such as CSF analyses and cerebral MRI scans were performed. Therefore, these reports are unable to support the described single observations further. A recently published autopsy series, available after the data cut-off date of this systematic study and thus not included in the results section of this manuscript, has shown that SARS-CoV-2 can be detected in the brain of 7 of 22 individuals studied [53]. This strongly supports neurotropism, warranting further assessment of its clinical relevance. The neuropathological features may be distinct from other viral encephalitides, as widespread microscopic infarcts and hemorrhagic white matter lesions were revealed, but not the prominent microglial nodules and perivascular inflammation, suggesting a vascular origin with secondary myelin loss during CNS inflammation [54–56].

Seizures in patients with COVID-19 might occur in consequence of direct brain infection, but only single reports of patients with seizures exist so far. Thus, current evidence does not suggest an additional risk of seizures in COVID-19 [41].

Encephalopathy, rather than encephalitis, may occur due to indirect mechanism of neuropathogenicity, such as hypoxic encephalopathy found in deceased COVID-19 patients [26]. In these cases, ARDS may act synergistically with intracranial hypertension, rendering the brain vulnerable to both amyloid-beta accumulation and cytokine-mediated hippocampal damage [57]. Hyper-inflammatory systemic responses may further contribute to neurological symptoms and rare but severe neurological complications. A focal parenchymal infiltrate of T lymphocytes above the normal range was detected in two autopsy cases [55]. This activated systemic immune response might ultimately lead to fatal encephalopathy or chronic CNS demyelination associated with long-term sequelae, depending on viral and host factors that may influence disease severity [17]. T-helper 1 cells producing IFN-γ and GM-CSF, previously reported in CNS neuroinflammation [20], have also been found in COVID-19 patients in intensive care units [21]. Furthermore, accumulating evidence suggests that severely affected COVID-19 patients might suffer from a cytokine storm syndrome, which has been implicated as the putative mechanism underlying a case of COVID-19 associated with acute necrotizing encephalopathy [46].

Concerning peripheral neurological immune-mediated complications, Gutiérrez-Ortiz et al. [49] reported two patients with Miller Fisher syndrome and polyneuritis cranialis in patients infected with SARS-CoV-2. Miller Fisher syndrome, a variant of Guillain–Barré syndrome, is an autoimmune disease that can manifest a few days to weeks following a viral upper respiratory or gastrointestinal infection. These reports may suggest that neurological complications of COVID-19 could occur as para-infectious autoimmune-mediated complications. Such complications are not specific for SARS-CoV-2, and currently available single reports do not suggest that the frequency is exceptionally high in COVID-19 patients.

Acute cerebrovascular events have been mostly observed in patients with severe or critical COVID-19 disease course [2]. Nevertheless, such associations are based on a limited number of cases and are irresolute, because patients with severe or complicated disease courses are more likely to suffer from relevant comorbidities, such as diabetes and hypertension. These factors portray independent risk factors for cerebrovascular diseases and connote a strong association bias. Moreover, glucose imbalances, believed to impact on the homeostasis of the brain, have been described in SARS-CoV-2-infected patients with diabetes [24]. Infection with SARS-CoV-2 might further drive dyslipidemia, which might associate with disease progression from mild to critical [25]. In severe or fatal COVID-19 cases, coagulopathy, including elevated D-dimer levels, prolonged prothrombin time, and decreased platelet counts have been highlighted in a recent meta-analysis [22]. Interestingly, hyper-fibrinolysis, as reflected by elevated serum D-dimer, was present in 97% of COVID-19 non-survivors at admission [58] and 71.4% of non-survivors met the criteria for disseminated intravascular coagulation [59]. For this reason, severely affected patients might also be more susceptible to cerebrovascular disease [2]. The other way round, patients with pre-existing cerebrovascular conditions, are more likely to have worse clinical outcomes after SARS-CoV-2 infection, possibly due to plasmin, a key player in fibrinolysis, contributing to enhanced virulence and pathogenicity of SARS-CoV-2 [60].

With this review, we sought to identify the neurological features of a SARS-CoV-2 infection and COVID-19. We found that frequently reported neurological symptoms comprise headache, dizziness, taste and smell dysfunctions, or impaired consciousness. These symptoms, however, are non-specific for infection with SARS-CoV-2. Taste and smell dysfunction may indicate neurotropism. However, reports on direct brain infection remain scarce. Risks for other more severe neurological complications, such as cerebrovascular disease including ischemic strokes, might be increased; systematic analysis so far is hindered by the low number of associated cases reported and known interactions of vascular risk factors with a severe or critical COVID-19 disease course.

Further studies will be needed to address whether neurological symptoms manifest due to direct infection of structures of the nervous system, constitute a reflection of a systemic inflammatory syndrome, or occur as a consequence of the higher prevalence of cardiovascular comorbidities. Even though reports of anosmia and few cases of encephalitis suggest a neurotropic potential of SARS-CoV-2, additional experimental studies are mandatory to confirm the pathophysiological mechanisms. In addition, the early involvement of neurologists in the treatment of patients with COVID-19, and standardized international registries, such as the *Lean European Open Survey for SARS-CoV-2 Infected Patients (LEOSS)* [61] with neurological items already integrated, will help to further elucidate the clinical relevance of this topic*.* Such registry may also include common neurologic disease as underlying condition to a larger extent than in the literature reviewed here, allowing to assess the possible influence of the SARS-CoV-2 infection on neurological conditions, and vice versa. In the absence of valid data, the pandemic caused considerable worries in a population with chronic neurological disability, such as in persons with multiple sclerosis, Parkinson’s disease, or dementia. However, it has not been demonstrated so far that a patient group with chronic neurological disease, but no cardiac, vascular, pulmonary, or metabolic disorder, is at higher risk of a less favorable outcome following a SARS-CoV-2 infection. Furthermore, although it has been speculated that individuals exposed to certain immunomodulatory or immunosuppressive therapy, e.g. for multiple sclerosis or brain tumors may experience a more severe COVID-19 disease course, evidence is lacking to support this assumption. Certain immunotherapies may even have the potential to protect from severe autoinflammatory reactions, and thus may even have beneficial effects, currently tested in clinical trials (https://clinicaltrials.gov/ct2/show/NCT04280588; https://clinicaltrials.gov/ct2/show/NCT04343768).

## Electronic supplementary material

Below is the link to the electronic supplementary material.

Supplementary file1 (DOCX 57 kb)

Supplementary file2 (DOCX 166 kb)

## Data Availability

All data included in this review are available in the articles listed in Online Resource Table III. Our search strategy for three Chinese databases and the studies excluded based on full text analysis are available on request.
