# Supplementary material for: A systematic review of neurological symptoms and complications of COVID-19
Source: J Neurol. 2020 Jul 20;268(2):392–402. doi: 10.1007/s00415-020-10067-3 (PMC7370630; doi:10.1007/s00415-020-10067-3)
Supplement: Supplementary file 1 — Supplementary file1 (DOCX 57 kb) [file 415_2020_10067_MOESM1_ESM.docx]

**Online Resource**

Neurological manifestations of COVID-19: A systematic review

Journal of Neurology

Xiangliang Chen, Sarah Laurent, Oezguer A. Onur, Nina N. Kleineberg, Gereon R. Fink, Finja Schweitzer, and Clemens Warnke

Corresponding Authors: Finja Schweitzer PhD and Clemens Warnke MD, University of Cologne, Faculty of Medicine and University Hospital Cologne, Department of Neurology, Kerpener Street 62, 50937 Cologne, Germany. E-mail: finja.schweitzer@uk-koeln.de; clemens.warnke@uk-koeln.de

**Table II: Summary of rare neurological manifestations described in case reports of COVID-19 patients**

|  | n/N | Study type - details | Brain imaging | CSF | Respiratory specimens | Ref |
| --- | --- | --- | --- | --- | --- | --- |
| Ischemic stroke | 11/221 | Case series - single-center consecutive patients | CT | - | throat swab (+) | Li et al., 2020^1^ |
|  | 3/13 | Case series - ARDS only | MRI | - | nasopharyngeal samples (+) | Helms et al., 2020^2^ |
|  | 1 | Case report - 85-y old male, admitted with a diagnosis of multiple cerebral infarctions, fever on in-hospital day 10, death on day 28 | Not provided | - | Positive, specimen not specified | 刘茜 et al.2020^3^ |
|  | 1 | Case report - 70-year-old male, secondary cerebral infarction after one-month treatment of critical COVID-19 | CT (hypoxic-ischemic encephalopathy, cerebral infarction and subarachnoid hemorrhage) | - | pharyngeal swab (+) | He et al., 2020^4^ |
| Cerebral hemorrhage | 1/221 | Case series - single-center consecutive patients | CT | - | throat swab (+) | Li et al., 2020^1^ |
| Cerebral sinus venous thrombosis | 1/221 | Case series - single-center consecutive patients | CT venography | - | throat swab (+) | Li et al., 2020^1^ |
| Meningoencephalitis | 1 | Case report - 41-y old female, new onset seizure, impaired consciousness and headache. No respiratory COVID-19 symptoms | CT (normal) | Leukocyte count 70 (100% lymphocytes), RBC 65, protein 100, glucose 120 (unit not given), SARS-COV-2 in CSF not tested | Positive, specimen not specified | Duong et al., 2020^5^ |
|  | 1 | Case report - 24-y old male with loss of consciousness and transient generalized seizure | MRI (right lateral ventriculitis and encephalitis mainly on right mesial temporal lobe and hippocampus) | Leukocyte count 12/µL (10 mononuclear, 2 polymorphonuclear), SARS-CoV-2 in CSF positive twice | nasopharyngeal swab (-) | Moriguchi et al., 2020^6^ |
|  | 1 | Case report - a male (age not provided) with impaired consciousness, meningeal irritation signs | CT (normal) | SARS-CoV-2 negative, laboratory tests within normal limits | Positive, specimen not specified | Ye et al., 2020^7^ |
|  | 1 | Case report, 56-y old man, ARDS and agitation on day 10, consciousness recovered on day 32 | CT (normal) | SARS-CoV-2 positive (registered in GISAID database, ICDC-DT005) | Not specified | Xiang et al., 2020^8^ |
| Tuberculous meningitis | 1 | Case report - 45-y old man, initial symptom of sore throat, transient loss of consciousness on day 7; stupor on day 27; death on day 35. | CT (hypodense lesions in bilateral basal ganglia, bilateral semi-oval center, and the left frontotemporal lobe) | SARS-CoV-2 was negative, gene X-pert mycobacterium tuberculosis was positive | nasopharyngeal swab (-)three times | Wang et al., 2020^9^ |
| Hypoxic encephalopathy | 23/113 | Case series of deceased patients | Not provided | Not provided | throat swab (+) | Chen et al., 2020^10^ |
| Acute hemorrhagic necrotizing encephalopathy | 1 | Case report - female (age not provided), 3-days history of cough, fever, and altered mental status | MR (hemorrhagic rim enhancing lesions within the bilateral thalami, medial temporal lobes, and subinsular regions) | SARS-CoV-2 not performed. HSV-1/2 and VZV-PCR negative. | nasopharyngeal swab (+) | Poyiadji et al., 2020^11^ |
| Steroid-responsive severe encephalopathy | 1 | Case report - 60-y old male, severe alterations of consciousness, akinetic mutism, severe apathy | CT/MRT (no specific alterations) | Negative for SARS-COV-2, herpesviruses, adenovirus; mild lymphocytic pleocytosis (18/uL), moderate protein elevation (696 mg/dl); OCB, autoimmune antibody neg | nasopharyngeal swab (+) | Pilotto et al., 2020^12^ |
| Acute Myelitis | 1 | Case report - 66-y-old male, paraparesis/paralysis, urinary and bowel incontinence | No MRI performed | CSF investigation not performed | nasopharyngeal swab (+) | Zhao et al., 2020^13^ |
| Seizure | 1/ 214 | Case series - multi-center consecutive patients | - | - | throat swab (+) | Mao et al., 2020^14^ |
| Guillain-Barré syndrome | 1 | Case report - 61-y-old female, acute and progressive weakness in both legs, typical electrophysiology findings | - | albuminocytologic dissociation | oropharyngeal swab (+) | Zhao et al., 2020^15^ |
| Miller Fisher Syndrome | 1 | Case report - 50-y old man; anosmia, ageusia, right internuclear ophthalmoparesis, right fascicular oculomotor palsy, ataxia, areflexia, positive testing for GD1b-IgG antibodies | - | SARS-COV-2 negative, albuminocytologic dissociation, infectious diagnostics negative | nasopharyngeal swab (+) | Gutiérrez-Ortiz et al., 2020^16^ |
| Polyneuritis cranialis | 1 | Case report - 39-y old man, ageusia, bilateral abducens palsy, areflexia, | - | SARS-COV-2 negative. White blood cell count = 2/μl (all monocytes), infectious diagnostics negative, albuminocytologic dissociation | nasopharyngeal swab (+) | Gutiérrez-Ortiz et al., 2020^16^ |
| Oculomotor nerve palsy | 1 | Case report - 62-year old male, persistent diplopia, and a droopy left eyelid; limb weakness | MRI (no specific alterations) | Not examined | throat swab (+) | Wei et al., 2020^17^ |

**References:**

1. Li Y, Wang M, Zhou Y, et al. Acute Cerebrovascular Disease Following COVID-19: A Single Center, Retrospective, Observational Study. Epub 2020 Mar 3. Accessed at: https://papers.ssrn.com/abstract=3550025. Accessed April 28, 2020.

2. Helms J, Kremer S, Merdji H, et al. Neurologic Features in Severe SARS-CoV-2 Infection [online]. N. Engl. J. Med. Massachusetts Medical Society; 2020. Accessed at: https://www.nejm.org/doi/full/10.1056/NEJMc2008597. Accessed April 27, 2020.

3. 刘茜, 王荣帅, 屈国强, et al. 新型冠状病毒肺炎死亡尸体系统解剖大体观察报告 - 临床指南汇编数据库. 法医学杂志. Chinese Medical Journals Publishing House Co., Ltd.; 2020;36:E034–E034.

4. He J, Cheng G, Xu W, Zhang L, Zeng Z. Diagnosis and treatment of an elderly patient with secondary cerebral infarction caused by COVID-19. J South Med Univ. 2020;40:351–352.

5. Duong L, Xu P, Liu A. Meningoencephalitis without Respiratory Failure in a Young Female Patient with COVID-19 Infection in Downtown Los Angeles, Early April 2020. Brain Behav Immun. Epub 2020 Apr.:S0889159120305092.

6. Moriguchi T, Harii N, Goto J, et al. A first case of meningitis/encephalitis associated with SARS-Coronavirus-2. Int J Infect Dis IJID Off Publ Int Soc Infect Dis. 2020;94:55–58.

7. M Y, Y R, T L. Encephalitis as a Clinical Manifestation of COVID-19 [online]. Brain. Behav. Immun. Brain Behav Immun; 2020. Accessed at: https://pubmed.ncbi.nlm.nih.gov/32283294/. Accessed April 27, 2020.

8. Xiang F, Xu X, Gao L, et al. First case of 2019 coronavirus disease with encephalitis. ChinaXiv. T202003.00015.

9. Wang L, Cai J, Luo H, et al. A case of coronavirus disease 2019 with tuberculous meningitis. Chin J Neurol. Chinese Medical Journals Publishing House Co., Ltd.; 2020;53:361–364.

10. Chen T, Wu D, Chen H, et al. Clinical characteristics of 113 deceased patients with coronavirus disease 2019: retrospective study. BMJ. Epub 2020 Mar 26.:m1091.

11. Poyiadji N, Shahin G, Noujaim D, Stone M, Patel S, Griffith B. COVID-19–associated Acute Hemorrhagic Necrotizing Encephalopathy: CT and MRI Features. Radiology [online serial]. Radiological Society of North America; Epub 2020 Mar 31. Accessed at: https://pubs.rsna.org/doi/abs/10.1148/radiol.2020201187. Accessed April 28, 2020.

12. Pilotto A, Odolini Si, Masciocchi S, et al. Steroid-responsive severe encephalopathy in SARS-CoV-2 infection [online]. Neurology; 2020 Apr. Accessed at: http://medrxiv.org/lookup/doi/10.1101/2020.04.12.20062646. Accessed April 30, 2020.

13. Zhao K, Huang J, Dai D, Feng Y, Liu L, Nie S. Acute myelitis after SARS-CoV-2 infection: a case report. [online]. Neurology; 2020 Mar. Accessed at: http://medrxiv.org/lookup/doi/10.1101/2020.03.16.20035105. Accessed March 27, 2020.

14. Mao L, Jin H, Wang M, et al. Neurologic Manifestations of Hospitalized Patients With Coronavirus Disease 2019 in Wuhan, China. JAMA Neurol [online serial]. Epub 2020 Apr 10. Accessed at: https://jamanetwork.com/journals/jamaneurology/fullarticle/2764549. Accessed April 19, 2020.

15. Zhao H, Shen D, Zhou H, Liu J, Chen S. Guillain-Barré syndrome associated with SARS-CoV-2 infection: causality or coincidence? Lancet Neurol. 2020;19:383–384.

16. Gutiérrez-Ortiz C, Méndez A, Rodrigo-Rey S, et al. Miller Fisher Syndrome and polyneuritis cranialis in COVID-19. Neurology [online serial]. Wolters Kluwer Health, Inc. on behalf of the American Academy of Neurology; Epub 2020 Apr 16. Accessed at: https://n.neurology.org/content/early/2020/04/17/WNL.0000000000009619. Accessed April 27, 2020.

17. Wei H, Yin H, Huang M, Guo Z. The 2019 novel cornoavirus pneumonia with onset of oculomotor nerve palsy: a case study. J Neurol. 2020;267:1550–1553.
