# Supplementary material for: A systematic review of neurological symptoms and complications of COVID-19
Source: J Neurol. 2020 Jul 20;268(2):392–402. doi: 10.1007/s00415-020-10067-3 (PMC7370630; doi:10.1007/s00415-020-10067-3)
Supplement: Supplementary file 2 — Supplementary file2 (DOCX 166 kb) [file 415_2020_10067_MOESM2_ESM.docx]

**Online Resource**

Neurological manifestations of COVID-19: A systematic review

Journal of Neurology

Xiangliang Chen, Sarah Laurent, Oezguer A. Onur, Nina N. Kleineberg, Gereon R. Fink, Finja Schweitzer, and Clemens Warnke

Corresponding Authors: Finja Schweitzer PhD and Clemens Warnke MD, University of Cologne, Faculty of Medicine and University Hospital Cologne, Department of Neurology, Kerpener Street 62, 50937 Cologne, Germany. E-mail: finja.schweitzer@uk-koeln.de; clemens.warnke@uk-koeln.de

**Table III: List of studies included in the systematic review for neurologic manifestations in COVID-19 patients^a^**

| Author  month year | Study type | Total sample size | Diagnostic criteria/laboratory confirmation method | Neurological manifestation [n/N (%)] | | | | | | Remark |
| --- | --- | --- | --- | --- | --- | --- | --- | --- | --- | --- |
|  |  |  |  | Headache | Dizziness | Smell dysfunction | Taste dysfunction | Impaired consciousness | Others |  |
| Chen et al^1^ Feb 2020^b^ | Retrospective hospital-based single-center case series | 99 | WHO interim guideline/confirmed by RT-PCR on throat-swab | 8/99 (8.1) |  |  |  | Confusion: 9/99 (9.1) |  |  |
| Chen et al.^2^ March 2020^b^ | Retrospective hospital-based single-center case series | 274 | Chinese guideline (6th edition)/confirmed by RT-PCR on throat swab | Total: 31/274 (11.3)  Survivors: 11/113 (9.7)  Non-survivors: 20/161 (12.4) | Total: 21/274 (7.7)  Survivors: 10/113 (8.8) Non-survivors: 20/161 (6.8) |  |  |  | Hypoxic encephalopathy 23/113 (20.4) | Data of survivors and non-survivors. Headache (total) excluded from table 1 due to overlap with Ref 15 (Qin et al.) |
| Deng et al.^3^ Feb 2020^b^ | Retrospective case series based on reports released by official channels | 41 | Not provided | 2/41 (4.9), no separate data for headache and dizziness | |  |  |  |  | Data of non-survivors. |
| Duong et al.^4^  April 2020^b^ | Case report | 1 | COVID-19 testing positive (not specified) | Yes |  |  |  | Yes | Meningoencephalitis presented with new onset seizure. EEG: Generalized slowing with no epileptic discharges. CSF: WBC: 70 (100% lymphocytes), RBC: 65, protein: 100, glucose: 120 (unit not given). | Rare neurological manifestation. CSF:  SARS-CoV-2 not tested. |
| Gutiérrez-Ortiz et al.^5^  April 2020^b^ | Case report | 2 | Confirmed by RT-PCR on oropharyngeal swab | Yes |  |  |  |  | 1 patient with Miller Fisher syndrome  1 patient with polyneuritis cranialis | Rare neurological manifestation. CSF:  SARS-CoV-2 negative (2/2) |
| Helms et al.^6^  April 2020^b^ | Retrospective hospital-based single-center case series | 58 | Confirmed by RT-PCR on nasopharyngeal samples |  |  |  |  | Agitation: 40/58 (69.0)  Confusion: 26/40 (65.0) | Corticospinal tract signs: 39/58. Dysexecutive syndrome: 15/45.  Brain MRI: leptomeningeal enhancement: 8/13, perfusion abnormalities: 11/11, cerebral ischemic stroke: 3/13.  EEG: nonspecific changes: 8/8.  CSF: oligoclonal bands with the same pattern in serum: 2/7, elevated CSF IgG and CSF protein levels: 1/7 | Data of ARDS patients. CSF:  SARS-CoV-2 negative (7/7) |
| Huang et al.^7^  Feb 2020^b^ | Retrospective hospital-based single-center case series | 41 | Confirmed by RT-PCR on lower respiratory tract specimens | Total: 3/38 (8.0)  ICU: 0/13  Non-ICU (counted as mild/moderate in table 1): 3/25 (12.0) |  |  |  |  |  | Data of ICU/non-ICU cases. Headache (total/ICU) excluded from table 1 due to overlap with Ref 1 (Chen et al.) and Ref 43 (Yang et al.) |
| Klopfenstein et al.^8^  April 2020^b^ | Retrospective hospital-based single-center case series | 114 | Confirmed by RT-PCR on respiratory samples, mainly  nasopharyngeal swabs, sputum, bronchial aspirates, or bronchoalveolar lavage fluids | 44/54 (81.5) of anosmia patients |  | Anosmia: 54/114 (47.4) |  |  |  | Headache data excluded from table 1, as it describes the prevalence in anosmia patients. |
| Lechien et al.^9^  April 2020^b^ | Prospective hospital-based multi-center case series | 417 | Confirmed by RT-PCR (not specified) |  |  | Total: 357/417 (85.6)  Anosmia: 284/417 (68.1)  Hyposmia: 73/417 (17.5), all mild/moderate | Total: 342/385 (88.8), all mild/moderate |  |  | Data of mild to moderate cases. |
| Liu et al.^10^  Feb 2020^b^ | Retrospective hospital-based multi-center case series | 137 | Confirmed by RT-PCR on sputum and nasopharyngeal swabs | 13/137 (9.5) |  |  |  |  |  |  |
| Lu et al.^11^  April 2020^b^ | Retrospective hospital-based multi-center case series | 304 | Chinese guideline (6th edition) |  |  |  |  |  | Neither acute symptomatic seizures nor status epilepticus were observed. | Evidence suggesting no additional risk of acute symptomatic seizures in people with COVID-19. |
| Mao et al.^12^  April 2020^b^ | Retrospective hospital-based multi-center case series | 214 | WHO interim guideline/confirmed by RT-PCR on throat swab | Total: 28/214 (13.1)  Severe: 15/88 (17.0)  Non-severe: 13/126 (10.3) | Total: 36/214 (16.8)  Severe: 17/88 (19.3)  Non-severe: 19/126 (15.1) | Total: 11/214 (5.1)  Severe: 3/88 (3.4)  Non-severe: 8/126 (6.3) | Total: 12/214 (5.6)  Severe: 3/88 (3.4)  Non-severe: 9/126 (7.1) | Total: 16/214 (7.5)  Severe: 13/88 (14.8)  Non-severe: 3/126 (2.4) | Acute cerebrovascular disease: 6/214 (2.8), 5 severe cases. Vision impairment: 3/214 (1.4), 2 severe cases. Nerve pain: 5/214 (2.3), 4 severe cases. Skeletal muscle injury: 23/214 (10.7), 17 severe cases. | Data separated by severe and non-severe cases. |
| Moein et al.^13^  April 2020^b^ | Hospital-based single-center case-control study | 60 patients and 60 age-, sex-matched controls | Confirmed by RT-PCR on nasopharyngeal wash/aspirate or nasal aspirate. |  |  | Anosmia: 59/60 (98.3) | Ageusia: 14/60 (23.3) |  |  |  |
| Moriguchi et al.^14^  March 2020^b^ | Case report | 1 | Confirmed by RT-PCR using CSF (not detected in the nasopharyngeal swab) | Yes |  |  |  | Loss of consciousness | Meningitis/encephalitis presented with generalized seizure. MRI: right lateral ventriculitis and encephalitis mainly on right mesial temporal lobe and hippocampus. CSF:12 cells/µl (mostly mononuclear). RT-PCR test for SARS-CoV-2 was positive twice. | Rare neurological manifestation. CSF:  SARS-CoV-2 positive. |
| Qin et al.^15^ March 2020^b^ | Retrospective hospital-based single-center case series | 452 | WHO interim guideline/confirmed by RT-PCR on nasal and pharyngeal swab | Total: 52/452 (11.5)  Severe: 39/286 (13.6)  Non-severe: 13/166 (7.8) |  |  |  |  |  | Data separated by severe and non-severe cases. |
| Tian et al.^16^ Feb 2020^b^ | Retrospective hospital-based multi-center case series | 262 | Chinese guideline (5th edition)/confirmed by RT-PCR on respiratory specimens | Total: 17/262 (6.5)  Severe: 3/46 (6.5)  Non-severe: 14/216 (6.5) |  |  |  |  |  | Data separated by severe and non-severe cases. |
| Yan et al. ^17^  April 2020^b^ | Internet-based single-institution cross-sectional study | 59 (and 203 patients with influenza-like symptoms) | PCR-confirmed testing for COVID-19 (not specified) | 39/59 (66.1),  Patients with influenza-like symptoms: 99/203 (48.8) |  | 40/59 (67.8)  Patients with influenza-like symptoms: 33/203 (16.3) | 42/59 (71.2)  Patients with influenza-like symptoms: 35/203 (17.2) |  |  | Data of patients with influenza-like symptoms but negative COVID-19 test. |
| Ye et al. ^18^  April 2020^b^ | Case report | 1 | Positive for SARS-CoV-2 (not specified) |  |  |  |  | Yes | Encephalitis with meningeal irritation signs (including nuchal rigidity, Kernig sign, and Brudzinski sign). CSF normal. RT-PCR in CSF test for SARS-CoV-2 negative. Head CT normal. | Rare neurological manifestation.  CSF: SARS-CoV-2 negative. |
| Zhang et al. ^19^  April 2020^b^ | Retrospective hospital-based single-center cohort study | 663 | WHO interim guideline and Chinese guideline (rev. 5th edition)/confirmed by RT-PCR on both nasal and pharyngeal swabs | Total: 20/663 (3.0)  Mild/moderate: 7/254  Severe: 11/315  Critical: 2/94  Survivors: 20/638  Non-survivors: 3/25 | Total: 23/663 (3.5)  Mild/moderate: 2/254  Severe: 15/315  Critical: 6/94  Survivors: 22/638  Non-survivors: 1/25 |  |  | Total: 10/663 (1.5)  Mild/moderate: 1/254  Severe: 0/315  Critical: 9/94  Survivors: 7/638  Non-survivors: 3/25 |  | Data separated by mild/moderate, severe and critical cases, and survivors and non-survivors. |
| Zhao et al. ^20^  April 2020^b^ | Case report | 1 | Confirmed by RT-PCR on oropharyngeal swab |  |  |  |  |  | Guillain-Barré syndrome | Rare neurological manifestation. Most probably secondary infection with SARS-CoV-2 |
| Chen et al.^21^  Feb 2020^c^ | Retrospective hospital-based single-center case series | 143 | Chinese guideline (5th edition)/ confirmed by RT-PCR on pharyngeal swab | Total: 28/143 (19.6)  Moderate: 18/107 (16.8)  Severe: 8/24 (33.3)  Critical: 2/12 (16.7) |  |  |  |  |  | Data separated by moderate, severe and critical cases. |
| COVID-19 National Incident Room Surveillance Team^22^  April 2020^c^ | Retrospective nationwide case series | 6606 | Not provided | 2134/5863 (36.4) |  |  |  |  |  |  |
| Dai et al.^23^  March 2020^c^ | Retrospective province-wide case series | 918 | Chinese guideline ( 5th edition) | 110/918 (12.0) |  |  |  |  |  |  |
| Fang et al.^24^  Feb 2020^c^ | Retrospective hospital-based single-center case series | 79 | Chinese guideline ( 6th edition)/confirmed by RT-PCR on sputum, nasopharyngeal swabs, or lower respiratory tract specimens | Total: 6/79 (7.6)  Moderate: 2/55 (3.6)  Severe/critical: 4/24 (16.7)   - No separate date for headache and dizziness | |  |  |  |  | Data separated by moderate and severe/critical cases. |
| Han et al.^25^ Feb 2020^c^ | Retrospective hospital-based single-center case series | 108 | Chinese guideline ( 5th edition)/confirmed by RT-PCR (not specified) | 14/108 (13.0), all mild |  |  |  |  |  | Data of mild cases. |
| He et al.^26^  March 2020^c^ | Case report | 1 | confirmed by RT-PCR on pharyngeal swab |  |  |  |  |  | Cerebral infarction presented with hypoxic-ischemic encephalopathy, cerebral infarction and subarachnoid hemorrhage after one month treatment of COVID-19. The patient died 2 days after the cerebrovascular event. | Rare neurological manifestation. |
| Li et al ^27^  March 2020^c^ | Retrospective hospital-based single-center case series | 221 | WHO interim guideline/confirmed by RT-PCR on throat swab |  |  |  |  |  | Acute ischemic stroke: 11/221. Cerebral venous sinus thrombosis: 1/221. Cerebral haemorrhage: 1/221 | Rare neurological manifestation. |
| Liu et al.^28^  Feb 2020^c^ | Retrospective hospital-based single-center case series | 61 | Chinese guideline ( 4th edition)/confirmed by RT-PCR on throat swab | Total: 21/61 (34.4)  Moderate: 18/44 (40.9)  Severe/critical: 3/17 (17.6) |  |  |  |  |  | Data separated by moderate and severe/critical cases. |
| 刘茜 et al. ^29^  March 2020^c^ | Autopsy study | 1 | Positive for SARS-CoV-2 (not specified) |  |  |  |  |  | Admitted with a diagnosis of multiple cerebral infarction. | Autopsy showed brain edema by gross examination without histopathology. |
| Lu et al.^30^  Feb 2020^c^ | Retrospective hospital-based single-center case series | 75 confirmed patients (and 124 patients with SARS-CoV-2 RT-PCR negative) | Chinese guideline ( 6th edition)/ confirmed by RT-PCR on nasal and pharyngeal swabs | 6/75 (8.8);  Patients SARS-CoV-2 RT-PCR negative: 3/124 (3.4) |  |  |  |  |  |  |
| Qiu et al.^31^ March 2020^c^ | Retrospective hospital-based multi-center case series | 36 | Chinese guideline ( 5th edition)confirmed by RT-PCR on upper nasopharyngeal swabs | Total: 3/36 (8.3)  Mild: 1/17 (5.9)  Moderate: 2/19 (10.5) |  |  |  |  |  | Data of children (0-16 years), separated by mild and moderate cases. |
| Shi et al.^32^  Feb 2020^c^ | Retrospective hospital-based multi-center case series | 81 | WHO interim guideline/confirmed by RT-PCR on throat swab | 5/81 (6.2) | 2/81 (2.5) |  |  |  |  | Headache (total) excluded from table 1, due to overlap with Ref 1 (Chen et al.) and Ref 12 (Mao et al.). |
| Spiteri et al.^33^  March 2020^c^ | Retrospective multi-country case series | 38 | confirmed by two separate SARS-CoV-2 RT-PCR tests | 6/38 (15.8) |  |  |  |  |  |  |
| Sun et al.^34^  April 2020^c^ | Retrospective hospital-based multi-center case series | 150 | Chinese guideline (rev. 5th edition)/confirmed by RT-PCR on sputum, nasopharyngeal swabs or lower respiratory tract specimens | 3/150 (2.0) |  |  |  |  |  |  |
| Wang et al.^35^  March 2020^c^ | Case report | 1 | Diagnosed according to Clinical and CT imaging features. RT-PCR on nasopharyngeal swab was negative for three times | Headache after recovery from the transient loss of consciousness. |  |  |  | Loss of consciousness for several minutes on day 7 after the initial symptom of sore throat; Fell into a stupor 20 days later; Dead after another 8 days. | Tuberculous meningitis presented with meningeal  irritation sign of nuchal rigidity; presence of bilateral Babinski signs; CSF: SARS-CoV-2 negative, gene X-pert mycobacterium tuberculosis positive; Head CT: low-density lesions in bilateral basal ganglia, bilateral semi-oval center and the left frontotemporal lobe | Rare neurological manifestation. CSF: SARS-CoV-2 negative. |
| Wei et al.^36^ Feb 2020^c^ | Case report | 1 | Confirmed by RT-PCR on throat swab |  |  |  |  |  | Oculomotor nerve palsy presented with persistent diplopia and a droopy left eyelid; limb weakness  and poor spirit. MRI: no new infarction, bleeding of brainstem or pituitary apoplexy, tumor and MS. MRA: no aneurysms. No indication for underlying structural cause of oculomotor nerve injury | Rare neurological manifestation. CSF: SARS-CoV-2 not tested. |
| Wen et al.^37^  Feb 2020^c^ | Retrospective hospital-based single-center case series | 46 | Chinese guideline (5th edition)/confirmed by RT-PCR on nasal or pharyngeal swabs | 15/46 （32.6）- no separate data for headache and dizziness | |  |  |  |  |  |
| Wu et al.^38^  Feb 2020^c^ | Retrospective hospital-based multi-center case series | 80 | WHO interim guideline/confirmed by RT-PCR on throat swab and/or nose swab | 13/80 (16.3) |  |  |  |  |  |  |
| Wu et al.^39^ March 2020^c^ | Retrospective hospital-based multi-center case series | 74 | WHO interim guideline/confirmed by RT-PCR on nasopharynx swab | 2/74 (2.7) |  |  |  |  |  | Data of children (0-16 years) |
| Xiang et al.^40^  March 2020^c^ | Case report | 1 | Confirmed by RT-PCR on CSF (RT-PCR on respiratory specimens not provided) |  | Dizziness as one of the initial symptoms. |  |  | Agitation (and ARDS) on the 10^th^ day, admitted to ICU. Consciousness recovered on day 32. | Viral encephalitis as clinical diagnosis. Generalized seizure developed on day 14; Meningeal irritation sign of nuchal rigidity; presence of bilateral Babinski signs; head CT normal; CSF: SARS-CoV-2 positive, genome was registered in GISAID database (ICDC-DT005). | Rare neurological manifestation. CSF: SARS-CoV-2 positive. |
| Xiang et al.^41^  March 2020^c^ | Retrospective hospital-based single-center case series | 49 | Chinese guideline (4th edition)/confirmed by RT-PCR on respiratory or blood samples | Total: 6/49 (12.2)  Moderate: 4/40 (10.0)  Severe/critical: 2/9 (22.2) – no separate data for headache and dizziness | |  |  |  |  | Data separated by moderate and severe/critical cases. |
| Xu et al.^42^ April 2020^c^ | Retrospective hospital-based multi-center case series | 32 | WHO interim guideline/confirmed by RT-PCR on throat swab | 2/21 (10.0) |  |  |  |  |  | Data of children (<18 years) |
| Yang et al.^43^  Feb 2020^c^ | Retrospective hospital-based single-center case series | 52 | WHO interim guideline, not further specified | Critical: 3/52 (5.8)  Survivors: 1/20 (5.0)  Non-survivors: 2/32 (6.3) |  |  |  |  |  | Data of severe cases, separated by survivors and non-survivors. |
| Yuan et al.^44^  March 2020^c^ | Retrospective citywide case series | 223 | Chinese guideline ( 6th edition)/confirmed by RT-PCR (samples not specified) | Total: 11/223 (4.9)  Mild/moderate: 10/192 (5.2)  Severe/critical: 1/31 (3.2) |  |  |  |  |  | Data separated by mild/moderate and severe/critical cases. |
| Ai et al.^45^  Feb 2020^d^ | Retrospective hospital-based single-center case series | 102 | Chinese guideline (5th edition)/confirmed by RT-PCR on throat swab |  | 4/102 (3.9) |  |  |  |  |  |
| Cao et al.^46^  March 2020^d^ | Retrospective citywide case series | 198 | Chinese guideline (5th edition)/confirmed by RT-PCR on throat swab | Total: 24/198 (12.1)  ICU: 0/19  Non-ICU: 24/179 (13.4), no separate data for headache and dizziness | |  |  |  |  | Data of ICU and non-ICU cases. |
| Chen et al.^47^  March 2020^d^ | Retrospective hospital-based single-center case series | 101 | Chinese guideline (5th edition)/ 56.4% were confirmed by RT-PCR | 3/101 (3.0) | 7/101 (6.9) |  |  |  |  | Data of non-survivors. |
| Chen et al.^48^  March 2020^d^ | Retrospective hospital-based multi-center case series | 534 | Chinese guideline (7th edition)/confirmed by RT-PCR on nasopharyngeal swabs | Mobile cabin hospital: 45/263 (17.1)  Tongji Hospital: 76/271 (28.0) |  |  |  |  |  | Headache (Tongji Hospital) excluded from calculations in table 1 due to overlap with Ref 15 (Qin et al.). |
| Chen et al.^49^ March 2020^d^ | Retrospective hospital-based single-center case series | 123 | Chinese guideline (6th edition)/confirmed by RT-PCR on throat swab | Total: 21/123 (17.1)  With HBV: 2/15 (13.3)  Without HBV: 19/108 (17.6) |  |  |  |  |  | Data of patients with and without HBV. |
| Chen et al.^50^  March 2020^d^ | Retrospective hospital-based multi-center case series | 291 | Chinese guideline (not specified)/confirmed by RT-PCR on throat swab | Total: 20/291 (6.9)  Mild: 2/29 (6.9)  Moderate: 13/212 (6.1)  Severe/critical: 5/50(10.0) | Total: 12/291 (4.1)  Mild: 1/29 (3.4)  Moderate: 10/212 (4.7)  Severe/critical: 1/50(2.0) |  |  |  |  | Data separated by mild, moderate and severe/critical cases. Headache (total) excluded from calculations in table 1 due to overlap with Ref 23 (Dai et al.). |
| Chen et al.^51^ April 2020^d^ | Retrospective hospital-based single-center case series | 284 | Chinese guideline (7th edition)/confirmed by RT-PCR on respiratory tract  samples | 32/284 (12.1) |  |  |  |  |  |  |
| Cummings et al.^52^ April 2020^d^ | Retrospective hospital-based multi-center case series | 257 | Confirmed by RT-PCR on naso- and/or oro-pharyngeal swab | 10/257 (3.9), all critical cases |  |  |  |  |  | Data of critical cases. |
| Fan et al.^53^ March 2020^d^ | Retrospective hospital-based single-center case series | 55 | Chinese guideline (5th edition) | Total: 2/55 (3.6)  Mild/moderate: 2/47 (4.3)  Severe/critical: 0/8 |  |  |  |  |  | Data separated by mild/moderate and severe/critical cases. |
| Feng et al.^54^  Feb 2020^d^ | Retrospective hospital-based multi-center case series | 141 | WHO interim guideline/confirmed by RT-PCR on nasal and pharyngeal swab | Total: 5/141 (3.5)  Stable: 4/126 (3.2)  Progressive: 1/15 (6.7),  all moderate |  |  |  |  |  | Data of moderate cases. Headache (total) excluded from table 1 due to overlap with Ref 23 (Dai et al.). |
| Fu et al.^55^ March 2020^d^ | Retrospective hospital-based single-center case series | 36 | Chinese guideline (5th edition)/confirmed by RT-PCR on respiratory or blood samples | 2/36 (5.6) |  |  |  |  |  |  |
| Guan et al.^56^  Feb 2020^d^ | Retrospective hospital-based multi-center case series | 1590 | WHO interim guideline/confirmed by RT-PCR on nasal and pharyngeal  swab | 205/1328 (15.4) |  |  |  | 20/1421 (1.4) |  |  |
| Guan et al.^57^  Feb 2020^d^ | Retrospective hospital-based multi-center case series | 1099 | WHO interim guideline/confirmed by RT-PCR on nasal and pharyngeal swab specimens | Total: 150/1099 (13.6)  Non-severe: 124/926 (13.4)  Severe: 26/173 (15.0) |  |  |  |  |  | Data of severe and non-severe cases available. Headache (total) excluded from table 1 due to overlap with Ref 56 (Guan et al.). |
| Guo et al.^58^ April 2020^d^ | Retrospective hospital-based multi-center case series | 159 | Chinese guideline (not specified) | 3/118 (2.5) |  |  |  |  |  | Data of non-survivors. |
| Hu et al.^59^  March 2020^d^ | Retrospective hospital-based single-center case series | 323 | WHO interim guideline and Chinese guideline (5th edition)/confirmed by RT-PCR on throat swab | Total: 3/323 (0.9)  Moderate: 3/151 (2.0)  Severe: 0/146  Critical: 0/26 |  |  |  |  |  | Data separated by moderate, severe and critical cases. |
| Huang et al.^60^ March 2020^d^ | Retrospective hospital-based single-center case series | 125 | Chinese guideline (6th edition) | Total: 24/125 (19.2)  Mild: 16/93 (17.2)  Severe: 8/32 (25.0) |  |  |  |  |  | Data separated by mild and severe cases. Headache (total) excluded from table 1 due to overlap with Ref 51 (Chen et al.). |
| Huang et al.^61^ March 2020^d^ | Retrospective hospital-based single-center case series | 36 | Confirmed by RT-PCR (not specified) |  |  |  |  | 8/36 (22.2) |  | Data of non-survivors. |
| Jiang et al.^62^ April 2020^d^ | Retrospective hospital-based single-center case series | 55 | WHO interim guideline and Chinese guideline (7th edition)/confirmed by RT-PCR on throat swab | Total: 10/55 (18.2)  Non-severe: 8/47 (17.0)  Severe: 2/8 (25.0) |  |  |  |  |  | Data separated by severe and non-severe cases. Headache (total) excluded from table 1 due to overlap with Ref 38 (Wu et al.). |
| Kluytmans-van den Bergh et al.^63^ March 2020^d^ | Retrospective hospital-based multi-center case series | 86 | Confirmed by RT-PCR on oropharyngeal samples | 49/86 (57.0) |  |  | 6/86 (7.0) |  |  | Data of healthcare workers. |
| Lei et al.^64^  March 2020^d^ | Retrospective hospital-based single-center case series | 67 | Confirmed by RT-PCR on nasal and pharyngeal swabs or blood samples | 9/67 (13.4), no separate data for headache and dizziness | |  |  |  |  |  |
| Leung et al.^65^ April 2020^d^ | Retrospective hospital-based multi-center case series | 50 | Confirmed by RT-PCR on sputum specimens and throat swabs pooled with nasopharyngeal aspirates |  |  |  |  | Confusion: 1/50 (2.0) |  |  |
| Levinson et al.^66^ April 2020^d^ | Retrospective hospital-based single-center case series | 45 | Confirmed by RT-PCR on naso-pharyngeal swabs | 20/42 (47.6), all mild | 9/42 (21.4), all mild | Anosmia: 15/42 (35.7), all mild | 14/42 (33.3), all mild |  |  | Data of mild cases. |
| Li et al.^67^  Feb 2020^d^ | Retrospective hospital-based single-center case series | 47 | Chinese guideline (5th edition)/confirmed by RT-PCR on throat swab | 3/47 (6.4), all severe |  |  |  |  |  | Data of severe cases. |
| Liu et al.^68^  Feb 2020^d^ | Retrospective hospital-based single-center case series | 51 | WHO interim guideline and Chinese guideline (5th edition) | Total: 5/51 (9.8)  Moderate: 5/44 (11.4)  Severe/critical: 0/7 | Total: 7/51 (13.7)  Moderate: 5/44 (11.4)  Severe/critical: 2/7 (28.6) |  |  |  |  | Data separated by moderate and severe/critical cases. |
| Liu et al.^69^  March 2020^d^ | Retrospective hospital-based single-center case series | 153 | Confirmed by RT-PCR on pharyngeal swab | 12/153 (7.8) | 4/153 (2.6) |  |  |  |  |  |
| Liu et al.^70^  March 2020^d^ | Retrospective hospital-based single-center case series | 41 | WHO interim guideline and Chinese guideline (5th edition)/confirmed by RT-PCR on throat swab | Total: 2/41 (4.9)  With CVM: 2/24 (8.3)  Without CVM: 0/17 | Total: 5/41 (12.2)  With CVM:2/24 (8.3)  Without CVM: 3/17 (17.6) |  |  |  |  | Data of healthcare workers, separated by patients with and without CVM. |
| Lu et al.^71^  Feb 2020^d^ | Retrospective citywide case series | 265 | WHO interim guideline and Chinese guideline (5th edition)/confirmed by RT-PCR on nasopharyngeal swab | Total: 26/265 (9.8)  Mild/moderate: 25/243 (10.3)  Severe/critical: 1/22 (4.5) |  |  |  |  |  | Data separated by mild/moderate and severe/critical cases. Headache (total) excluded from table 1 due to overlap with Ref 87 (Zeng et al.). |
| Nie et al.^72^  March 2020^d^ | Retrospective hospital-based single-center case series | 97 | Chinese guideline (5th edition)/confirmed by RT-PCR on nasopharyngeal and anal swab samples | Total: 7/97 (7.2)  Mild: 5/72 (6.9)  Severe: 2/25 (8.0), no separate data for headache and dizziness | |  |  |  |  | Data separated by mild and severe cases. |
| Pilotto et al.^73^ April 2020^d^ | Case report | 1 | Confirmed by RT-PCR on nasopharyngeal swab |  |  |  |  | Severe alterations of consciousness; progressive irritability, confusion and asthenia. | Steroid-responsive severe encephalopathy presented as akinetic mutism with a wakeful state of profound apathy. Brain CT: unremarkable. Brain MRI with gadolinium: no significant alterations. EEG: generalized slowing. CSF: HSV-1, HSV-2, HSV-6, HSV-8, EBV, VZV, Adenovirus, SARS-CoV-2 negative; mild lymphocytic pleocytosis (18/uL), moderate CSF protein (696 mg/dl); no oligoclonal bands. No sign of immune-mediated encephalitis (NMDAR, LGI1, CASPR2, GABAAR, GABABR and AMPAR, Ri, Yo, Ma2, Hu, Amphiphysin negative), anti-MOG antibodies negative | Rare neurological manifestation. CSF: SARS-CoV-2 negative. |
| Poyiadji et al.^74^  March 2020^d^ | Case report | 1 | Confirmed by RT-PCR on nasopharyngeal swab |  |  |  |  |  | Acute hemorrhagic necrotizing encephalopathy with a 3-day history of cough, fever, and altered mental status. MRI: hemorrhagic rim enhancing lesions within the bilateral thalami, medial temporal lobes, and subinsular regions. SARS-CoV-2-PCR in blood positive. HSV-1/2 and VZV-PCR in CSF neg. Further CSF analysis was not performed. | Rare neurological manifestation. No CSF data available. |
| Qi et al.^75^  March 2020^d^ | Retrospective hospital-based multi-center case series | 267 | WHO interim guideline and severity categorized by American Thoracic Society guideline/confirmed by RT-PCR on nasopharyngeal swab and bronchoalveolar lavage fluid, or anal swab |  |  |  |  | Confusion: Total: 25/267 (9.4)  Non-severe: 15/217 (6.9)  Severe: 10/50 (20) |  | Data separated by severe and non-severe cases. |
| Shi et al.^76^  April 2020^d^ | Retrospective hospital-based single-center case series | 101 | WHO interim guideline/confirmation method not specified | 5/101 (5.0) |  |  |  |  |  | Data of non-survivors. |
| Tabata et al.^77^  April 2020^d^ | Retrospective hospital-based single-center case series | 104 | Confirmed by RT-PCR on the pharyngeal swab or sputum | 10/104 (9.6) |  |  |  |  |  |  |
| Tian et al.^78^  March 2020^d^ | Retrospective hospital-based single-center case series | 37 | Chinese guideline (5th and 6th edition)/confirmed by RT-PCR on nasal and pharyngeal swabs | 3/37 (8.1) |  |  |  |  |  |  |
| Wang et al.^79^ April 2020^d^ | Retrospective hospital-based single-center case series | 45 | Chinese guideline (6th edition)/confirmed by RT-PCR | Total: 4/45 (8.9)  Moderate: 4/35 (11.4)  Severe: 0/10 |  |  |  |  |  | Data separated by moderate and severe cases. |
| Wen et al.^80^  March 2020^d^ | Retrospective citywide case series | 417 | WHO interim guideline and Chinese guideline (6th edition)/confirmed by RT-PCR on sputum, blood, broncho-alveolar lavage fluid, naso- or oro-pharyngeal swab | Total: 55/417 (13.2)  Mild/moderate: 52/381 (13.6)  Severe/critical: 3/36 (8.3) |  |  |  |  |  | Data separated by mild/moderate and severe/critical cases. |
| Xi et al.^81^  March 2020^d^ | Retrospective hospital-based single-center case series | 788 | WHO interim guideline/confirmed by RT-PCR on throat swab and sputum | 66/722 (8.4) |  |  |  |  |  |  |
| Xu et al.^82^  Feb 2020^d^ | Retrospective citywide case series | 434 | Confirmed patients without specific definition | 39/434 (9.0) |  |  |  |  |  |  |
| Xu et al.^83^  March 2020^d^ | Retrospective hospital-based multi-center case series | 69 | WHO interim guideline/confirmed by RT-PCR on pharyngeal swab | Total: 23/69 (33.3)  Mild: 18/44 (40.9)  Severe/critical: 5/25 (20.0) |  |  |  |  |  | Data of patients without comorbidities, data separated by mild and severe/critical cases. |
| Yan et al.^84^  March 2020^d^ | Retrospective hospital-based single-center case series | 168 | WHO interim guideline and Chinese guideline (3rd edition)/confirmed by RT-PCR on sputum and throat swab | Total: 14/168 (8.3)  Non-severe: 9/132 (6.8)  Severe: 5/36 (13.9) | Total: 12/168 (7.1)  Non-severe: 8/132 (6.1)  Severe: 4/36 (11.1) |  |  |  |  | Data separated by severe and non-severe cases. |
| Yang et al.^85^ April 2020^d^ | Retrospective hospital-based single-center case-control study | 126 confirmed patients with hypertension, 125 non-hypertension | Chinese guideline (5th edition)/confirmed method not specified | Hypertension: 20/126 (15.9)  Non-hypertension: 15/125 (12.1) |  |  |  |  |  | Data of patients with and without hypertension. |
| Yang et al.^86^  March 2020^d^ | Retrospective hospital-based single-center case series | 55 | Chinese guideline (6th edition)/confirmed by RT-PCR on respiratory sample | Total: 6/55 (10.9)  With pneumonia: 2/21 (9.5)  Without pneumonia: 4/34 (11.8) | Total: 9/55 (16.4)  With pneumonia: 6/ 21 (28.6)  Without pneumonia: 3/34 (8.8) |  |  |  |  | Data of patients with and without pneumonia. |
| Zeng et al.^87^  March 2020^d^ | Retrospective hospital-based multi-center case series | 752 | Confirmed by RT-PCR on pharyngeal swab | Shanghai: 33/338 (9.8)  Bejing: 46/414 (11.1) |  |  |  |  |  |  |
| Zhang et al.^88^  Feb 2020^d^ | Retrospective hospital-based single-center case series | 82 | Confirmed by RT-PCR on nasal and pharyngeal swab |  |  |  |  | 17/82 (20.7) |  | Data of non-survivors. |
| Zhang et al.^89^  March 2020^d^ | Retrospective hospital-based single-center case series | 221 | WHO interim guideline/confirmed by RT-PCR on pharyngeal swab | Total: 17/221 (7.7)  Non-severe: 13/166 (7.8)  Severe: 4/55 (7.3) |  |  |  |  |  | Data separated by severe and non-severe cases. |
| Zhao et al.^90^  April 2020^d^ | Case report | 1 | Confirmed by RT-PCR on nasopharyngeal swab |  |  |  |  |  | Acute myelitis presented with weakness of both lower limbs with urinary and bowel incontinence, culminating in flaccid lower extremity paralysis. CSF and MRI analysis were not performed. | Rare neurological manifestation. CSF: SARS-CoV-2 not tested. |
| Zhao et al.^91^  March 2020^d^ | Retrospective hospital-based single-center case series | 77 | WHO interim guideline/confirmed by RT-PCR on nasopharyngeal swab | Total: 10/77 (13)  Non-severe: 10/57 (17.5)  Severe: 0/20, no separate data for headache and dizziness | |  |  |  |  | Data separated by severe and non-severe cases. |
| Zheng et al.^92^ April 2020^d^ | Retrospective hospital-based single-center case series | 34 | WHO interim guideline/confirmed by RT-PCR on respiratory specimen, | Total: 2/34 (5.9)  IMV: 2/15 (13.3)  NIV: 0/19 |  |  |  |  |  | Data of ICU cases. |

^a^Data are n/N (%),where N is the total number of patients with available data. WHO interim guideline is the World Health Organization interim guidance for clinical management when COVID-19 is suspected (https://www.who.int/publications-detail/clinical-management-of-severe-acute-respiratory-infection-when-novel-coronavirus-(ncov)-infection-is-suspected). Chinese guidelines are developed by the National Health Committee of the People's Republic of China (http://www.nhc.gov.cn/). Literature is reported in alphabetical order of first author, organized by pubmed retrievals (^b^), other sources (^c^) and preprints (^d^).

Abbreviations: CVM, cardiovascular manifestation; NIV, non-invasive ventilation; IMV, invasive mechanical ventilation

**References:**

1. Chen N, Zhou M, Dong X, et al. Epidemiological and clinical characteristics of 99 cases of 2019 novel coronavirus pneumonia in Wuhan, China: a descriptive study. The Lancet. 2020;395:507–513.

2. Chen T, Wu D, Chen H, et al. Clinical characteristics of 113 deceased patients with coronavirus disease 2019: retrospective study. BMJ. Epub 2020 Mar 26.:m1091.

3. Deng S-Q, Peng H-J. Characteristics of and Public Health Responses to the Coronavirus Disease 2019 Outbreak in China. J Clin Med. 2020;9:575.

4. Duong L, Xu P, Liu A. Meningoencephalitis without Respiratory Failure in a Young Female Patient with COVID-19 Infection in Downtown Los Angeles, Early April 2020. Brain Behav Immun. Epub 2020 Apr.:S0889159120305092.

5. Gutiérrez-Ortiz C, Méndez A, Rodrigo-Rey S, et al. Miller Fisher Syndrome and polyneuritis cranialis in COVID-19. Neurology [online serial]. Wolters Kluwer Health, Inc. on behalf of the American Academy of Neurology; Epub 2020 Apr 16. Accessed at: https://n.neurology.org/content/early/2020/04/17/WNL.0000000000009619. Accessed April 27, 2020.

6. Helms J, Kremer S, Merdji H, et al. Neurologic Features in Severe SARS-CoV-2 Infection [online]. N. Engl. J. Med. Massachusetts Medical Society; 2020. Accessed at: https://www.nejm.org/doi/full/10.1056/NEJMc2008597. Accessed April 27, 2020.

7. Huang C, Wang Y, Li X, et al. Clinical features of patients infected with 2019 novel coronavirus in Wuhan, China. Lancet Lond Engl. 2020;395:497–506.

8. Klopfenstein T, Kadiane-Oussou NJ, Toko L, et al. Features of anosmia in COVID-19. Médecine Mal Infect. Epub 2020 Apr.:S0399077X20301104.

9. Jr L, Cm C-E, Dr DS, et al. Olfactory and Gustatory Dysfunctions as a Clinical Presentation of Mild-To-Moderate Forms of the Coronavirus Disease (COVID-19): A Multicenter European Study [online]. Eur. Arch. Oto-Rhino-Laryngol. Off. J. Eur. Fed. Oto-Rhino-Laryngol. Soc. EUFOS Affil. Ger. Soc. Oto-Rhino-Laryngol. - Head Neck Surg. Eur Arch Otorhinolaryngol; 2020. Accessed at: https://pubmed.ncbi.nlm.nih.gov/32253535/. Accessed April 27, 2020.

10. Liu K, Fang Y-Y, Deng Y, et al. Clinical characteristics of novel coronavirus cases in tertiary hospitals in Hubei Province: Chin Med J (Engl). Epub 2020 Feb.:1.

11. Lu L, Xiong W, Liu D, et al. New‐onset acute symptomatic seizure and risk factors in Corona Virus Disease 2019: A Retrospective Multicenter Study. Epilepsia. Epub 2020 Apr 18.:epi.16524.

12. Mao L, Wang M, Chen S, et al. Neurological Manifestations of Hospitalized Patients with COVID-19 in Wuhan, China: a retrospective case series study [online]. Infectious Diseases (except HIV/AIDS); 2020 Feb. Accessed at: http://medrxiv.org/lookup/doi/10.1101/2020.02.22.20026500. Accessed March 27, 2020.

13. Moein ST, Hashemian SMR, Mansourafshar B, Khorram‐Tousi A, Tabarsi P, Doty RL. Smell dysfunction: a biomarker for COVID‐19. Int Forum Allergy Rhinol. Epub 2020 Apr 17.:alr.22587.

14. Moriguchi T, Harii N, Goto J, et al. A first case of meningitis/encephalitis associated with SARS-Coronavirus-2. Int J Infect Dis IJID Off Publ Int Soc Infect Dis. 2020;94:55–58.

15. Qin C, Zhou L, Hu Z, et al. Dysregulation of immune response in patients with COVID-19 in Wuhan, China. Clin Infect Dis [online serial]. Epub 2020 Mar 12. Accessed at: https://academic.oup.com/cid/article/doi/10.1093/cid/ciaa248/5803306. Accessed April 19, 2020.

16. Tian S, Hu N, Lou J, et al. Characteristics of COVID-19 infection in Beijing. J Infect. 2020;80:401–406.

17. Yan CH, Faraji F, Prajapati DP, Boone CE, DeConde AS. Association of chemosensory dysfunction and Covid‐19 in patients presenting with influenza‐like symptoms. Int Forum Allergy Rhinol [online serial]. John Wiley & Sons, Ltd; Epub 2020 Apr 12. Accessed at: https://onlinelibrary.wiley.com/doi/10.1002/alr.22579. Accessed April 23, 2020.

18. M Y, Y R, T L. Encephalitis as a Clinical Manifestation of COVID-19 [online]. Brain. Behav. Immun. Brain Behav Immun; 2020. Accessed at: https://pubmed.ncbi.nlm.nih.gov/32283294/. Accessed April 27, 2020.

19. Zhang J, Wang X, Jia X, et al. Risk factors for disease severity, unimprovement, and mortality of COVID-19 patients in Wuhan, China. Clin Microbiol Infect. Epub 2020 Apr.:S1198743X20302172.

20. Zhao H, Shen D, Zhou H, Liu J, Chen S. Guillain-Barré syndrome associated with SARS-CoV-2 infection: causality or coincidence? Lancet Neurol. 2020;19:383–384.

21. Chen Y, Shui L, Pang X, et al. Clinical features of coronavirus disease 2019 in Northeast area of Chongqing: analysis of 143 cases. Chunhui. 2020;42:549–554.

22. COVID-19 National Incident Room Surveillance Team. COVID-19, Australia: Epidemiology Report 12: Reporting week ending 23:59 AEST 19 April 2020. Commun Dis Intell [online serial]. 2020;44. Accessed at: https://www1.health.gov.au/internet/main/publishing.nsf/Content/1D03BCB527F40C8BCA258503000302EB/$File/covid_19_australia_epidemiology_report_12_reporting_week_ending_23_59_aest_19_april_2020.pdf. Accessed April 28, 2020.

23. Dai Z, Gao L, Luo K, et al. Clinical characteristics analysis of novel coronavirus pneumonia in Hunan province. Pract Prev Med [online serial]. 2020;27. Accessed at: https://kns.cnki.net/KCMS/detail/43.1223.R.20200305.1537.005.html?uid=WEEvREcwSlJHSldRa1FhdkJtNEYwbVZYZDdmWWpDNXJzZHlyMXU3RlhYVT0=$9A4hF_YAuvQ5obgVAqNKPCYcEjKensW4ggI8Fm4gTkoUKaID8j8gFw!!&v=MDA5MTA5QlpPc0xZdzlNem1SbjZqNTdUM2ZscVdNMENMTDdSN3FkWitacUZpM2xXN3pNSUZjPU5qVFNkN0c0SE5ITXJJ. Accessed May 5, 2020.

24. Fang X, Mei Q, Yang T, et al. Clinical characteristics and treatment strategies of 79 patients with COVID-19. Chin Pharmacol Bull. 2020;36:453–459.

25. Han R, Huang L, Jiang H, Dong J, Peng H, Zhang D. Early Clinical and CT Manifestations of Coronavirus Disease 2019 (COVID-19) Pneumonia. Am J Roentgenol. Epub 2020 Mar 17.:1–6.

26. He J, Cheng G, Xu W, Zhang L, Zeng Z. Diagnosis and treatment of an elderly patient with secondary cerebral infarction caused by COVID-19. J South Med Univ. 2020;40:351–352.

27. Li Y, Wang M, Zhou Y, et al. Acute Cerebrovascular Disease Following COVID-19: A Single Center, Retrospective, Observational Study. Epub 2020 Mar 3. Accessed at: https://papers.ssrn.com/abstract=3550025. Accessed April 28, 2020.

28. Liu J, Liu Y, Xiang P, et al. Neutrophil-to-Lymphocyte Ratio Predicts Severe Illness Patients with 2019 Novel Coronavirus in the Early Stage. medRxiv. Cold Spring Harbor Laboratory Press; Epub 2020 Feb 12.:2020.02.10.20021584.

29. 刘茜, 王荣帅, 屈国强, et al. 新型冠状病毒肺炎死亡尸体系统解剖大体观察报告 - 临床指南汇编数据库. 法医学杂志. Chinese Medical Journals Publishing House Co., Ltd.; 2020;36:E034–E034.

30. Lu J, Hu S, Fan R, et al. ACP risk grade: a simple mortality index for patients with confirmed or suspected severe acute respiratory syndrome coronavirus 2 disease (COVID-19) during the early stage of outbreak in Wuhan, China. medRxiv. Cold Spring Harbor Laboratory Press; Epub 2020 Feb 23.:2020.02.20.20025510.

31. Qiu H, Wu J, Hong L, Luo Y, Song Q, Chen D. Clinical and epidemiological features of 36 children with coronavirus disease 2019 (COVID-19) in Zhejiang, China: an observational cohort study. Lancet Infect Dis. Epub 2020 Mar.:S1473309920301985.

32. Shi H, Han X, Jiang N, et al. Radiological findings from 81 patients with COVID-19 pneumonia in Wuhan, China: a descriptive study. Lancet Infect Dis. Elsevier; 2020;20:425–434.

33. Spiteri G, Fielding J, Diercke M, et al. First cases of coronavirus disease 2019 (COVID-19) in the WHO European Region, 24 January to 21 February 2020. Eurosurveillance [online serial]. 2020;25. Accessed at: https://www.eurosurveillance.org/content/10.2807/1560-7917.ES.2020.25.9.2000178. Accessed May 5, 2020.

34. Sun C, Zhang XB, Dai Y, Xu XZ, Zhao J. Clinical analysis of 150 cases of 2019 novel coronavirus infection in Nanyang City, Henan Province. Chin J Tuberc Respir Dis. 2020;43:E042.

35. Wang L, Cai J, Luo H, et al. A case of coronavirus disease 2019 with tuberculous meningitis. Chin J Neurol. Chinese Medical Journals Publishing House Co., Ltd.; 2020;53:361–364.

36. Wei H, Yin H, Huang M, Guo Z. The 2019 novel cornoavirus pneumonia with onset of oculomotor nerve palsy: a case study. J Neurol. 2020;267:1550–1553.

37. Wen K, Li W, Zhang D, et al. Epidemiological and clinical characteristics of 46 newly-admitted coronavirus disease 2019 cases in Beijing. Chin J Infect Dis [online serial]. 2020;38. Accessed at: http://rs.yiigle.com/yufabiao/1182702.htm. Accessed April 29, 2020.

38. Wu J, Liu J, Zhao X, et al. Clinical Characteristics of Imported Cases of COVID-19 in Jiangsu Province: A Multicenter Descriptive Study. Clin Infect Dis Off Publ Infect Dis Soc Am [online serial]. Oxford University Press; Accessed at: https://www.ncbi.nlm.nih.gov/pmc/articles/PMC7108195/. Accessed April 28, 2020.

39. Wu Q, Xing Y, Shi L, et al. Epidemiological and Clinical Characteristics of Children with Coronavirus Disease 2019 [online]. Epidemiology; 2020 Mar. Accessed at: http://medrxiv.org/lookup/doi/10.1101/2020.03.19.20027078. Accessed May 5, 2020.

40. Xiang F, Xu X, Gao L, et al. First case of 2019 coronavirus disease with encephalitis. ChinaXiv. T202003.00015.

41. Xiang T, Liu J, Xu F, et al. Analysis of clinical characteristics of 49 patients with coronavirus disease 2019 in Jiangxi. Chin J Respir Crit Care Med. 华西期刊社; 2020;19:154–160.

42. Xu H, Liu E, Xie J, et al. A follow-up study of children infected with SARS-CoV-2 from Western China [online]. Infectious Diseases (except HIV/AIDS); 2020 Apr. Accessed at: http://medrxiv.org/lookup/doi/10.1101/2020.04.20.20073288. Accessed May 5, 2020.

43. Yang X, Yu Y, Xu J, et al. Clinical course and outcomes of critically ill patients with SARS-CoV-2 pneumonia in Wuhan, China: a single-centered, retrospective, observational study. Lancet Respir Med [online serial]. Elsevier; Accessed at: https://www.ncbi.nlm.nih.gov/pmc/articles/PMC7102538/. Accessed April 28, 2020.

44. 袁婧, 孙艳雨, 左玉洁, et al. 重庆市223例新型冠状病毒肺炎患者的临床特征分析. 西南大学学报(自然科学版). 2020;42:17–24.

45. Ai J, Chen J, Wang Y, et al. The cross-sectional study of hospitalized coronavirus disease 2019 patients in Xiangyang, Hubei province [online]. Infectious Diseases (except HIV/AIDS); 2020 Feb. Accessed at: http://medrxiv.org/lookup/doi/10.1101/2020.02.19.20025023. Accessed April 29, 2020.

46. Cao M, Zhang D, Wang Y, et al. Clinical Features of Patients Infected with the 2019 Novel Coronavirus (COVID-19) in Shanghai, China [online]. Respiratory Medicine; 2020 Mar. Accessed at: http://medrxiv.org/lookup/doi/10.1101/2020.03.04.20030395. Accessed March 27, 2020.

47. Chen Ji, Fan H, Zhang L, et al. Retrospective Analysis of Clinical Features in 101 Death Cases with COVID-19 [online]. Intensive Care and Critical Care Medicine; 2020 Mar. Accessed at: http://medrxiv.org/lookup/doi/10.1101/2020.03.09.20033068. Accessed March 27, 2020.

48. Chen L, Deng C, Chen X, et al. Ocular manifestations and clinical characteristics of 534 cases of COVID-19 in China: A cross-sectional study [online]. Ophthalmology; 2020 Mar. Accessed at: http://medrxiv.org/lookup/doi/10.1101/2020.03.12.20034678. Accessed March 27, 2020.

49. Chen X, Jiang Q, Ma Z, et al. Clinical Characteristics of Hospitalized Patients with SARS-CoV-2 and Hepatitis B virus Co-infection. medRxiv. Epub 2020.:2020.03.23.20040733.

50. Chen X, Zheng F, Qing Y, et al. Epidemiological and clinical features of 291 cases with coronavirus disease 2019 in areas adjacent to Hubei, China: a double-center observational study [online]. Respiratory Medicine; 2020 Mar. Accessed at: http://medrxiv.org/lookup/doi/10.1101/2020.03.03.20030353. Accessed March 27, 2020.

51. Chen X, Zhang Y, Zhu B, et al. Associations of clinical characteristics and antiviral drugs with viral RNA clearance in patients with COVID-19 in Guangzhou, China: a retrospective cohort study [online]. Infectious Diseases (except HIV/AIDS); 2020 Apr. Accessed at: http://medrxiv.org/lookup/doi/10.1101/2020.04.09.20058941. Accessed April 30, 2020.

52. Cummings MJ, Baldwin MR, Abrams D, et al. Epidemiology, clinical course, and outcomes of critically ill adults with COVID-19 in New York City: a prospective cohort study [online]. Intensive Care and Critical Care Medicine; 2020 Apr. Accessed at: http://medrxiv.org/lookup/doi/10.1101/2020.04.15.20067157. Accessed April 30, 2020.

53. Fan L, Liu C, Li N, et al. Medical treatment of 55 patients with COVID-19 from seven cities in northeast China who fully recovered: a single-center, retrospective, observational study [online]. Respiratory Medicine; 2020 Mar. Accessed at: http://medrxiv.org/lookup/doi/10.1101/2020.03.28.20045955. Accessed May 1, 2020.

54. Feng Z, Yu Q, Yao S, et al. Early Prediction of Disease Progression in 2019 Novel Coronavirus Pneumonia Patients Outside Wuhan with CT and Clinical Characteristics [online]. Infectious Diseases (except HIV/AIDS); 2020 Feb. Accessed at: http://medrxiv.org/lookup/doi/10.1101/2020.02.19.20025296. Accessed March 27, 2020.

55. Fu H, Li H, Tang X, et al. Analysis on the Clinical Characteristics of 36 Cases of Novel Coronavirus Pneumonia in Kunming [online]. Infectious Diseases (except HIV/AIDS); 2020 Mar. Accessed at: http://medrxiv.org/lookup/doi/10.1101/2020.02.28.20029173. Accessed March 27, 2020.

56. Guan W, Liang W, Zhao Y, et al. Comorbidity and its impact on 1,590 patients with COVID-19 in China: A Nationwide Analysis [online]. Respiratory Medicine; 2020 Feb. Accessed at: http://medrxiv.org/lookup/doi/10.1101/2020.02.25.20027664. Accessed March 27, 2020.

57. Guan W, Ni Z, Hu Y, et al. Clinical characteristics of 2019 novel coronavirus infection in China [online]. Respiratory Medicine; 2020 Feb. Accessed at: http://medrxiv.org/lookup/doi/10.1101/2020.02.06.20020974. Accessed March 27, 2020.

58. Guo A-X, Cui J-J, OuYang Q-Y, He L, Guo C-X, Yin J-Y. The clinical characteristics and mortal causes analysis of COVID-19 death patients [online]. Infectious Diseases (except HIV/AIDS); 2020 Apr. Accessed at: http://medrxiv.org/lookup/doi/10.1101/2020.04.12.20062380. Accessed April 30, 2020.

59. Hu L, Chen S, Fu Y, et al. Risk Factors Associated with Clinical Outcomes in 323 COVID-19 Patients in Wuhan, China [online]. Infectious Diseases (except HIV/AIDS); 2020 Mar. Accessed at: http://medrxiv.org/lookup/doi/10.1101/2020.03.25.20037721. Accessed March 27, 2020.

60. Huang H, Cai S, Li Y, et al. Prognostic factors for COVID-19 pneumonia progression to severe symptom based on the earlier clinical features: a retrospective analysis [online]. Infectious Diseases (except HIV/AIDS); 2020 Mar. Accessed at: http://medrxiv.org/lookup/doi/10.1101/2020.03.28.20045989. Accessed May 1, 2020.

61. Huang Y, Yang R, Xu Y, Gong P. Clinical characteristics of 36 non-survivors with COVID-19 in Wuhan, China [online]. Infectious Diseases (except HIV/AIDS); 2020 Feb. Accessed at: http://medrxiv.org/lookup/doi/10.1101/2020.02.27.20029009. Accessed April 29, 2020.

62. Jiang X, Tao J, Wu H, et al. Clinical features and management of severe COVID-19: A retrospective study in Wuxi, Jiangsu Province, China [online]. Infectious Diseases (except HIV/AIDS); 2020 Apr. Accessed at: http://medrxiv.org/lookup/doi/10.1101/2020.04.10.20060335. Accessed April 30, 2020.

63. Kluytmans M, Buiting A, Pas S, et al. SARS-CoV-2 infection in 86 healthcare workers in two Dutch hospitals in March 2020. medRxiv. Epub 2020.:2020.03.23.20041913.

64. Lei Y, lan yunping, lu jianli, huang xiaobo, silang bamu, zeng fan. Clinical features of imported cases of coronavirus disease 2019 in Tibetan patients in the Plateau area [online]. Infectious Diseases (except HIV/AIDS); 2020 Mar. Accessed at: http://medrxiv.org/lookup/doi/10.1101/2020.03.09.20033126. Accessed March 27, 2020.

65. Leung KS-S, Ng TT-L, Wu AK-L, et al. A territory-wide study of early COVID-19 outbreak in Hong Kong community: A clinical, epidemiological and phylogenomic investigation [online]. Infectious Diseases (except HIV/AIDS); 2020 Mar. Accessed at: http://medrxiv.org/lookup/doi/10.1101/2020.03.30.20045740. Accessed May 1, 2020.

66. Levinson R, Elbaz M, Ben-Ami R, et al. Anosmia and dysgeusia in patients with mild SARS-CoV-2 infection [online]. Infectious Diseases (except HIV/AIDS); 2020 Apr. Accessed at: http://medrxiv.org/lookup/doi/10.1101/2020.04.11.20055483. Accessed April 30, 2020.

67. Li J, Zhang Y, Wang F, et al. Sex differences in clinical findings among patients with coronavirus disease 2019 (COVID-19) and severe condition [online]. Respiratory Medicine; 2020 Feb. Accessed at: http://medrxiv.org/lookup/doi/10.1101/2020.02.27.20027524. Accessed March 27, 2020.

68. lei liu, Jian-ya G. Clinical characteristics of 51 patients discharged from hospital with COVID-19 in Chongqing，China [online]. Infectious Diseases (except HIV/AIDS); 2020 Feb. Accessed at: http://medrxiv.org/lookup/doi/10.1101/2020.02.20.20025536. Accessed March 27, 2020.

69. Liu L, Liu W, Wang S, Zheng S. A preliminary study on serological assay for severe acute respiratory syndrome coronavirus 2 (SARS-CoV-2) in 238 admitted hospital patients [online]. Infectious Diseases (except HIV/AIDS); 2020 Mar. Accessed at: http://medrxiv.org/lookup/doi/10.1101/2020.03.06.20031856. Accessed March 27, 2020.

70. Liu R, Ming X, Xu O, et al. Association of Cardiovascular Manifestations with In-hospital Outcomes in Patients with COVID-19: A Hospital Staff Data [online]. Infectious Diseases (except HIV/AIDS); 2020 Mar. Accessed at: http://medrxiv.org/lookup/doi/10.1101/2020.02.29.20029348. Accessed March 27, 2020.

71. Lu H, Ai J, Shen Y, et al. A descriptive study of the impact of diseases control and prevention on the epidemics dynamics and clinical features of SARS-CoV-2 outbreak in Shanghai, lessons learned for metropolis epidemics prevention [online]. Infectious Diseases (except HIV/AIDS); 2020 Feb. Accessed at: http://medrxiv.org/lookup/doi/10.1101/2020.02.19.20025031. Accessed March 27, 2020.

72. Nie S, Zhao X, Zhao K, Zhang Z, Zhang Z, Zhang Z. Metabolic disturbances and inflammatory dysfunction predict severity of coronavirus disease 2019 (COVID-19): a retrospective study [online]. Infectious Diseases (except HIV/AIDS); 2020 Mar. Accessed at: http://medrxiv.org/lookup/doi/10.1101/2020.03.24.20042283. Accessed March 27, 2020.

73. Pilotto A, Odolini Si, Masciocchi S, et al. Steroid-responsive severe encephalopathy in SARS-CoV-2 infection [online]. Neurology; 2020 Apr. Accessed at: http://medrxiv.org/lookup/doi/10.1101/2020.04.12.20062646. Accessed April 30, 2020.

74. Poyiadji N, Shahin G, Noujaim D, Stone M, Patel S, Griffith B. COVID-19–associated Acute Hemorrhagic Necrotizing Encephalopathy: CT and MRI Features. Radiology [online serial]. Radiological Society of North America; Epub 2020 Mar 31. Accessed at: https://pubs.rsna.org/doi/abs/10.1148/radiol.2020201187. Accessed April 28, 2020.

75. Qi D, Yan X, Tang X, et al. Epidemiological and clinical features of 2019-nCoV acute respiratory disease cases in Chongqing municipality, China: a retrospective, descriptive, multiple-center study [online]. Respiratory Medicine; 2020 Mar. Accessed at: http://medrxiv.org/lookup/doi/10.1101/2020.03.01.20029397. Accessed March 27, 2020.

76. Shi Q, Zhao K, Yu J, et al. Clinical characteristics of 101 non-surviving hospitalized patients with COVID-19: A single center, retrospective study [online]. Infectious Diseases (except HIV/AIDS); 2020 Mar. Accessed at: http://medrxiv.org/lookup/doi/10.1101/2020.03.04.20031039. Accessed March 27, 2020.

77. Tabata S, Imai K, Kawano S, et al. Non-severe vs severe symptomatic COVID-19: 104 cases from the outbreak on the cruise ship “Diamond Princess” in Japan [online]. Infectious Diseases (except HIV/AIDS); 2020 Mar. Accessed at: http://medrxiv.org/lookup/doi/10.1101/2020.03.18.20038125. Accessed March 27, 2020.

78. Tian S, Chang Z, Wang Y, et al. Clinical characteristics and reasons of different duration from onset to release from quarantine for patients with COVID-19 Outside Hubei province, China. [online]. Infectious Diseases (except HIV/AIDS); 2020 Mar. Accessed at: http://medrxiv.org/lookup/doi/10.1101/2020.03.21.20038778. Accessed March 27, 2020.

79. wang changzheng, Li C. Preliminary study to identify severe from moderate cases of COVID-19 using NLR&RDW-SD combination parameter [online]. Infectious Diseases (except HIV/AIDS); 2020 Apr. Accessed at: http://medrxiv.org/lookup/doi/10.1101/2020.04.09.20058594. Accessed April 30, 2020.

80. Wen Y, Wei L, Li Y, et al. Epidemiological and clinical characteristics of COVID-19 in Shenzhen, the largest migrant city of China [online]. Infectious Diseases (except HIV/AIDS); 2020 Mar. Accessed at: http://medrxiv.org/lookup/doi/10.1101/2020.03.22.20035246. Accessed March 27, 2020.

81. Xi J, Xu K, Jiang P, et al. Virus strain of a mild COVID-19 patient in Hangzhou representing a new trend in SARS-CoV-2 evolution related to Furin cleavage site [online]. Infectious Diseases (except HIV/AIDS); 2020 Mar. Accessed at: http://medrxiv.org/lookup/doi/10.1101/2020.03.10.20033944. Accessed March 27, 2020.

82. Xu L, Yuan J, Zhang Y, et al. Highland of COVID-19 outside Hubei: epidemic characteristics, control and projections of Wenzhou, China [online]. Epidemiology; 2020 Feb. Accessed at: http://medrxiv.org/lookup/doi/10.1101/2020.02.25.20024398. Accessed March 27, 2020.

83. Xu Y, Li Y, Zeng Q, et al. Clinical Characteristics of SARS-CoV-2 Pneumonia Compared to Controls in Chinese Han Population [online]. Infectious Diseases (except HIV/AIDS); 2020 Mar. Accessed at: http://medrxiv.org/lookup/doi/10.1101/2020.03.08.20031658. Accessed March 27, 2020.

84. Yan S, Song X, Lin F, et al. Clinical Characteristics of Coronavirus Disease 2019 in Hainan, China [online]. Infectious Diseases (except HIV/AIDS); 2020 Mar. Accessed at: http://medrxiv.org/lookup/doi/10.1101/2020.03.19.20038539. Accessed March 27, 2020.

85. Yang G, Tan Z, Zhou L, et al. Angiotensin II Receptor Blockers and Angiotensin-Converting Enzyme Inhibitors Usage is Associated with Improved Inflammatory Status and Clinical Outcomes in COVID-19 Patients With Hypertension [online]. Infectious Diseases (except HIV/AIDS); 2020 Apr. Accessed at: http://medrxiv.org/lookup/doi/10.1101/2020.03.31.20038935. Accessed May 1, 2020.

86. Yang P, Ding Y, Xu Z, et al. Epidemiological and clinical features of COVID-19 patients with and without pneumonia in Beijing, China [online]. Epidemiology; 2020 Mar. Accessed at: http://medrxiv.org/lookup/doi/10.1101/2020.02.28.20028068. Accessed March 27, 2020.

87. Zeng Q, Li Y, Huang G, Wu W, Dong S, Xu Y. Mortality of COVID-19 is Associated with Cellular Immune Function Compared to Immune Function in Chinese Han Population [online]. Infectious Diseases (except HIV/AIDS); 2020 Mar. Accessed at: http://medrxiv.org/lookup/doi/10.1101/2020.03.08.20031229. Accessed March 27, 2020.

88. Zhang B, Zhou X, Qiu Y, et al. Clinical characteristics of 82 death cases with COVID-19 [online]. Infectious Diseases (except HIV/AIDS); 2020 Feb. Accessed at: http://medrxiv.org/lookup/doi/10.1101/2020.02.26.20028191. Accessed April 29, 2020.

89. Zhang G, Hu C, Luo L, et al. Clinical features and outcomes of 221 patients with COVID-19 in Wuhan, China [online]. Respiratory Medicine; 2020 Mar. Accessed at: http://medrxiv.org/lookup/doi/10.1101/2020.03.02.20030452. Accessed March 27, 2020.

90. Zhao K, Huang J, Dai D, Feng Y, Liu L, Nie S. Acute myelitis after SARS-CoV-2 infection: a case report. [online]. Neurology; 2020 Mar. Accessed at: http://medrxiv.org/lookup/doi/10.1101/2020.03.16.20035105. Accessed March 27, 2020.

91. Zhao W, Yu S, Zha X, et al. Clinical characteristics and durations of hospitalized patients with COVID-19 in Beijing: a retrospective cohort study [online]. Infectious Diseases (except HIV/AIDS); 2020 Mar. Accessed at: http://medrxiv.org/lookup/doi/10.1101/2020.03.13.20035436. Accessed March 27, 2020.

92. Zheng Y, Sun L, Xu M, et al. Clinical characteristics of 34 COVID-19 patients admitted to ICU in Hangzhou, China [online]. Infectious Diseases (except HIV/AIDS); 2020 Apr. Accessed at: http://medrxiv.org/lookup/doi/10.1101/2020.04.12.20062604. Accessed April 30, 2020.
